# Single-cell RNA-seq reveals cell type-specific molecular and genetic associations with primary open-angle glaucoma

**DOI:** 10.1038/s41392-025-02438-x

**Published:** 2025-10-10

**Authors:** Huaping Tian, Yuhong Chen, Tujing Zhao, Lin Ye, Hongjing Li, Zheng Li, Wenqiao Qiu, Wentao Wang, Runze Li, Fulin Liu, Haojue Xue, Ruilin Liao, Chao Qu, Jie Li, Liang Zou, Yi Shi, Zhenglin Yang, Lulin Huang

**Affiliations:** 1 Genetic Diseases Key Laboratory of Sichuan Province, Center for Medical Genetics, Department of Laboratory Medicine, Sichuan Academy of Medical Sciences and Sichuan Provincial People’s Hospital, School of Medicine, University of Electronic Science and Technology of China, Chengdu, China; 2 Department of Ophthalmology and Visual Science, Eye and Ear Nose Throat Hospital, Shanghai Medical School, Fudan University, Shanghai, China; 3 Department of Ophthalmology, Sichuan Provincial People’s Hospital, University of Electronic Science and Technology of China, Chengdu, China; 4 School of Food and Biological Engineering, Chengdu University, Chengdu, Sichuan China; 5 Sichuan-Chongqing Joint Key Laboratory for Pathology and Laboratory Medicine, Jinfeng Laboratory, Chongqing, China

**Keywords:** Neurodevelopmental disorders, Neurological disorders

## Abstract

Primary open-angle glaucoma (POAG), a leading cause of irreversible blindness, involves complex neurodegeneration in which the contribution of systemic immunity remains enigmatic. Here, we dissect the circulating immune landscape in POAG patients via high-resolution single-cell RNA sequencing of ~1.4 million peripheral blood mononuclear cells (PBMCs) from 110 patients and 110 controls of Chinese ancestry. We revealed significant immune remodeling in POAG, characterized by increased CD4^+^ T lymphocytes and myeloid cells and impaired cytolytic potential, as evidenced by reduced cell proportions of terminally differentiated CD8^+^ GZMK^+^ T cells and NK cells. Transcriptomic analysis revealed a sophisticated dual transcriptional landscape in which both proinflammatory and neuroprotective signaling pathways coexist across multiple immune cell lineages. While TNF and IFNG pathway genes were broadly downregulated, specific inflammatory activation components and neuroprotective genes were upregulated in distinct cell populations, suggesting that POAG represents a complex immunometabolic syndrome characterized by a dysregulated balance between inflammatory and neuroprotective signaling. Cell type-specific eQTL mapping and SMR analysis revealed that POAG genetic risk loci exert their effects through immune gene regulation in specific PBMC subsets. Functional validation using *Ifng*^*-/-*^ and *Tnf*^*+/-*^ mice in an LPS/NMDA-induced retinal injury model, which mirrored the immune alterations observed in human POAG, demonstrated that genetic deficiency in these pathways markedly exacerbated retinal ganglion cell loss and visual pathway deficits. Our study establishes a crucial link between systemic immune dysregulation—specifically the disrupted balance between inflammatory and neuroprotective signaling—and retinal health, highlighting the importance of restoring this balance for future POAG therapeutic strategies.

## Introduction

Glaucoma is a common local neurodegenerative disorder affecting the visual system and remains a major global cause of irreversible blindness. As one of the leading causes of irreversible blindness worldwide,^1,2^ glaucoma affects more than 70 million individuals, with approximately 10% experiencing bilateral blindness, underscoring its profound public health impact.^3^ Adult glaucoma is broadly classified into primary open-angle glaucoma (POAG) and primary angle-closure glaucoma (PACG), reflecting distinct anatomical and pathophysiological mechanisms.^1^ POAG, in particular, is characterized by progressive retinal ganglion cell (RGC) death and degeneration of optic nerve axons, culminating in irreversible visual field loss.^4^ Recent genetic studies have identified various genes associated with POAG, including *VAV2*, which plays a role in cytoskeletal organization and trabecular meshwork function.^5^ However, the role of systemic immune dysregulation in POAG pathogenesis remains poorly understood. Although intraocular pressure (IOP)-related stress remains a central risk factor in POAG, the elevation of intraocular pressure primarily results from impaired aqueous humor outflow through the trabecular meshwork, where increased resistance to fluid drainage leads to progressive pressure buildup within the anterior chamber. However, convergent evidence suggests that elevated IOP cannot fully account for the observed neuronal vulnerability and clinical heterogeneity in POAG, indicating that systemic factors and tissue-specific responses play crucial roles in determining disease severity and progression patterns.^4^ An imbalance between neuroinflammatory and neuroprotective mechanisms may contribute to neurologic injury.^6–8^ This emerging understanding of neuroinflammatory processes motivates a systems-level investigation of systemic and tissue-specific immune processes that may influence POAG risk, progression, and therapeutic response.

Historically, the eye has been considered an immune-privileged organ. Recently, lymphatic or lymphatic-like vessels have been shown to contribute to several ocular pathologies at various peri- and intraocular locations.^9^ Many genes participate in the immunological processes of glaucoma in eye tissues^10,11^ or the gut environment.^12^ Bulk transcriptomic profiling implicated increased proinflammatory cytokines in glaucoma eye tissues as a hallmark of the disease.^13^ In addition, the presence of IgG antibodies against retinal antigens in glaucoma indicates the involvement of an autoimmune mechanism in the development of glaucoma.^14–20^ Significant increases in aqueous humor IL-8^21^ and serum IL-4^22^ were detected in POAG patients, suggesting that abnormal immune environments may contribute to glaucomatous neuropathy in POAG patients. Quantified composition on the basis of known cell surface markers recently revealed B and T-cell alterations in POAG via flow cytometry analyses^23–25^ and revealed the neutrophil-to-lymphocyte ratio as a novel biomarker of POAG.^26–29^ Collectively, these findings provide convergent clues that imbalances in immune mediators measured in the blood may initiate, mirror, or exacerbate disease processes in POAG and therefore warrant systematic assessment.^29,30^

Despite these advances, no transcriptomic analyses of peripheral blood mononuclear cells (PBMCs) in POAG have been reported to date, leaving a critical gap in our understanding of systemic immune perturbations at single-cell resolution. Single-cell RNA sequencing (scRNA-seq) of PBMCs offers a comprehensive and unbiased platform to simultaneously profile cellular compositions and cell type-specific transcriptional states across circulating immune populations.^30,31^ When integrated with genome-wide genotyping, scRNA-seq enables the mapping of cis- and trans-expression quantitative trait loci (eQTLs), thereby linking genetic variation to context-dependent gene regulation in specific immune cell types. Importantly, cell type-specific cis-eQTLs are enriched for heritability across complex traits, highlighting their relevance to disease biology and genetic architecture.^32^ This matters particularly in glaucoma, where many regulatory effects are tissue^33–35^ and cell type-specific,^36–38^ such that disease-relevant genetic influences may only be detectable in the appropriate cellular context or activation state. Integrating eQTL maps with genome-wide association study (GWAS) data further refines causal inference, facilitates fine-mapping of disease-associated variants, and helps identify effector genes and the immune cell types through which they act.^39,40^ In the absence of such integrated, cell-resolved datasets in POAG, the capacity to connect systemic immune signatures with genetic predisposition—and to nominate therapeutic targets—is necessarily constrained. This integrative framework also enables the nomination of circulating biomarkers and immune cell states that may facilitate patient stratification and prospective risk prediction in clinical settings.

A crucial knowledge gap therefore persists regarding whether and how systemic immune alterations observed in peripheral blood directly influence retinal vulnerability in POAG. Establishing the functional consequences of specific immune pathways in human-relevant contexts is paramount for moving beyond association to mechanism. To address this unmet need, we leverage high-throughput scRNA-seq of PBMCs from a large cohort (*n* = 210) of POAG patients and healthy controls, coupled with genome-wide genotyping, to construct a high-resolution atlas of the molecular and genetic landscape of circulating immunity in POAG. We quantify cell type compositions and delineate disease-associated transcriptional programs, while performing comprehensive cis- and trans-eQTL mapping to illuminate genetic control of immune gene expression across relevant immune subsets. We further integrate these regulatory maps with POAG GWAS using summary-data Mendelian randomization (SMR) to prioritize putative causal genes and pinpoint the immune cell contexts in which risk variants exert their effects. Finally, to bridge systemic observations with local pathology, we functionally validate the impact of key downregulated pathways—specifically TNF and IFN-γ—via targeted genetic deletion in mouse models subjected to experimental retinal injury. Together, this integrated strategy offers new insights into the interplay among systemic immunity, genetic predisposition, and retinal neurodegeneration in POAG, establishes an important resource, and provides a mechanistically grounded perspective for precision risk stratification and therapeutic intervention for POAG.

## Results

### Single-cell atlas reveals immune cell remodeling in POAG

**Fig. 1:**
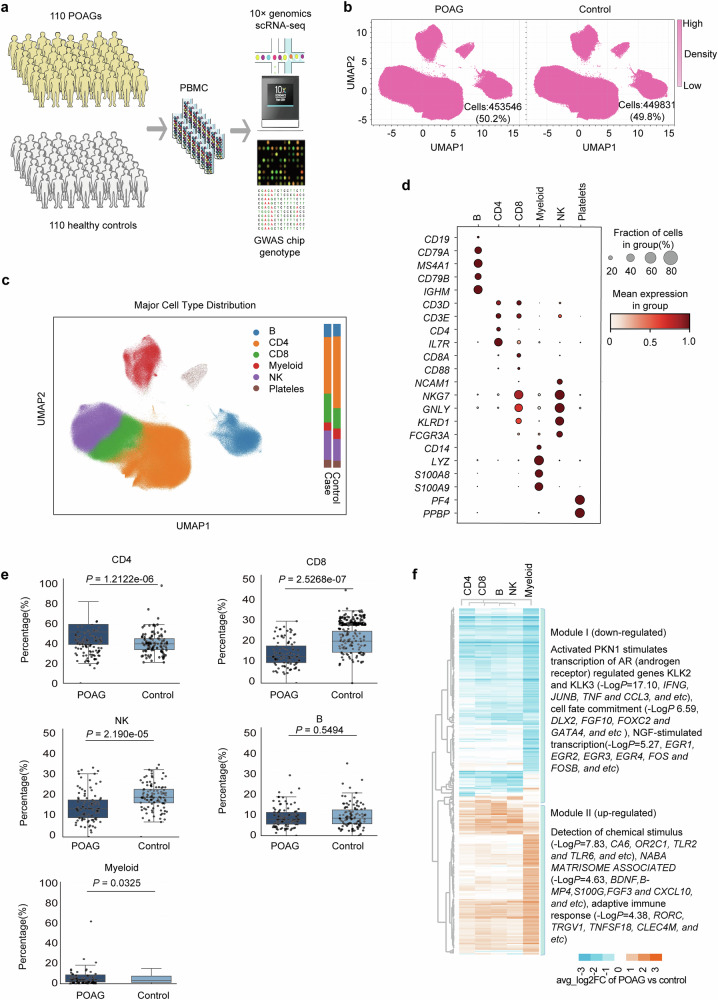
Single-cell transcriptomic profiling revealed altered peripheral immune cell composition and gene expression in POAG patients. **a** Schematic overview of the study design: peripheral blood mononuclear cells (PBMCs) were collected from 110 primary open-angle glaucoma (POAG) patients and 110 healthy controls, followed by 10x Genomics single-cell RNA sequencing (scRNA-seq) and genome-wide genotyping. **b** UMAP density plots showing the distribution of major immune cell populations in PBMCs from POAG patients and healthy controls. **c** UMAP visualization of single-cell transcriptomes, colored by major immune cell types (CD4^+^ T cells, CD8^+^ T cells, myeloid cells, NK cells, and B cells) and platelets. A total of 4129 platelets were removed from downstream analyses. The bar plots on the right summarize the relative proportions of each cell type in the POAG and control groups. **d** Dot plot displaying the expression of canonical marker genes used to identify each immune cell population. The dot size represents the fraction of cells expressing the gene, and the color indicates the average expression level. **e** Box plots comparing the proportions of each major immune cell type between POAG patients and controls. *P* values were calculated via appropriate statistical tests. **f** Heatmap of the top 300 differentially expressed genes (DEGs) across major immune cell types, comparing POAG patients to controls. Two main gene modules are highlighted: Module I (downregulated in POAG, enriched for PKN1 signaling and cell fate commitment genes) and Module II (upregulated in POAG, enriched for genes involved in chemical stimulus detection and adaptive immune responses)

To comprehensively define the circulating immune landscape in POAG, we performed 10x Genomics scRNA-seq on PBMCs from 110 POAG patients and 110 matched healthy controls of Chinese Han ancestry (Fig. 1a). A separate library was created for each of the 220 samples, and a total of 1,416,923 cells in the 220 samples were sequenced. Among them, 98 POAG patients and 107 healthy controls passed the final quality control (Supplementary Tables 1 and 2). After quality control and doublet removal using Scrublet, red blood cells (RBCs) with 200 > UMI > 10,000 genes and cells that lost typical PBMC markers were removed; the final dataset contained a total of 903,377 high-quality cells and 36,598 genes. Louvain clustering^41^ analysis of normalized and sample-corrected single-cell transcriptomic profiles suggested that there were no observable obvious batch effects on the distribution of cells between POAG patients and controls (Fig. 1b). These cells were subsequently identified on the basis of specific markers.^42^ We employed a rigorous cell type identification pipeline combining classical clustering approaches with well-established lineage markers. Using the Leiden algorithm with multiple resolution parameters, we identified six major cell populations (Fig. 1c, d). Cell type annotation was based on canonical markers: *CD3D* (T cells), *CD4*/*CD8A* (T cell subsets), *NCAM1* (NK cells), *CD19* (B cells), and *CD14* (myeloid cells). To ensure annotation accuracy, we performed comprehensive differential expression analysis for marker validation and cross-referenced our annotations with established immunology literature. Platelets were subsequently removed from downstream analyses. We then assessed differences in the cellular composition between the POAG patients and healthy controls by comparing the percentages of the five cell types. Compared with healthy controls, POAG patients presented significant alterations in immune cell composition, with increased cell proportions of CD4^+^ T cells (*P* = 1.21 × 10^−6^) and myeloid cells (*P* = 0.033) and decreased cell proportions of CD8^+^ T cells (*P* = 2.53 × 10^−7^) and NK cells (*P* = 2.19 × 10^−5^) (Fig. 1e, Supplementary Table 3). B lymphocytes did not significantly differ in terms of cell composition between the POAG patients and healthy controls. The significantly increased CD4^+^ T cell proportion and decreased CD8^+^ T cell proportion in POAG patients are consistent with previous observations.^24^

We then investigated the top 300 genes that were differentially expressed (DEGs) in at least one cell type between the POAG patients and healthy controls (|log(fold change)| >0.5; *P*_adjusted_ < 0.05; Fig. 1f, Supplementary Table 4).^39^ This expression heatmap of the five PBMC types with RNA expression data suggest that lymphoid cells (CD4^+^ T, CD8^+^ T, NK and B) are closely related and separate from myeloid cells (Fig. 1f). This finding was consistent with previous findings.^39^ Hierarchical clustering of the gene expression profiles of these DEGs across cell types resulted in two modules (Fig. 1f). Module I: Compared with healthy controls, POAG patients presented downregulated expression of modules of signaling pathways such as activated PKN1, which stimulates the transcription of the AR (androgen receptor)-regulated genes *KLK2* and *KLK3* (−log10 of *P*_adjusted_ = 17.10, including *IFNG*, *JUNB*, *TNF* and *CCL3*); cell fate commitment (−log10 of *P*_adjusted_ = 6.59, including *DLX2*, *FGF10*, *FOXC2* and *GATA4*); and NGF-stimulated transcription (−log10 of *P*_adjusted_ = 5.27, including *EGR1*, *EGR2*, *EGR3, EGR4*, *FOS* and *FOSB*). Module II: Compared with healthy controls, POAG patients presented upregulated expression of modules of signaling pathways such as those associated with chemical stimuli (−log10 of *P*_adjusted_ = 7.83, including *CA6*, *OR2C1, TLR2* and *TLR6*) and NABA MATRISOME ASSOCIATED (−log10 of *P*_adjusted_ = 4.63, including *BDNF, BMP4, S100G, FGF3* and *CXCL10*). This initial analysis revealed distinct pathway alterations in POAG PBMCs.

### Subpopulation analysis highlights dysregulation within PBMC compartments

**Fig. 2:**
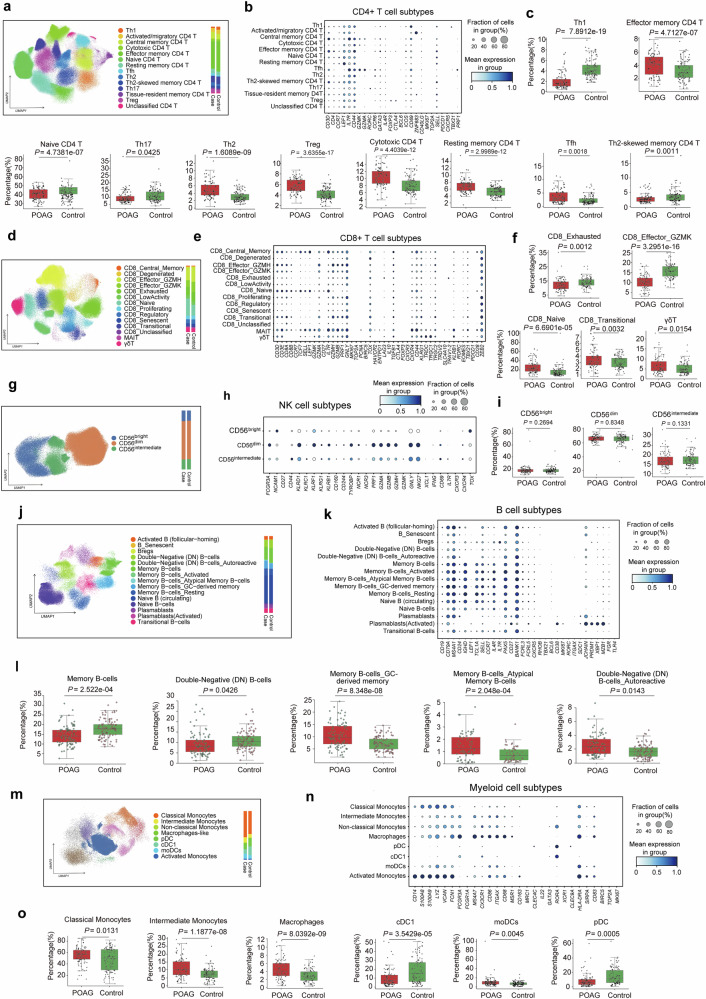
Single-cell transcriptomic profiling reveals altered immune cell subtype compositions in POAG. **a**, **d**, **g**, **j**, **m** UMAP plots showing clustering and annotation of major PBMC lineages and their subtypes, including CD4⁺ T, CD8⁺ T, NK, B, and myeloid cells from POAG patients and healthy controls. **b**, **e**, **h**, **k**, **n** Dot plots displaying canonical marker gene expression for each immune cell subtype. **c**, **f**, **i**, **l**, **o** Box plots comparing the proportions of subtypes with significant differences between groups (*P* values indicated); subtypes without significant differences are not shown. POAG patients present increases in exhausted and transitional CD8⁺ T cells, memory and autoreactive B cells, classical/intermediate monocytes, and macrophages (monocytes with macrophage-like characteristics), with reductions in dendritic cell subsets. These results highlight broad immune dysregulation and shifts in both adaptive and innate immune compartments in POAG

To investigate more granular cell identities, we analyzed five broad cell populations—CD4^+^ T, CD8^+^ T, NK, B and myeloid cells (Fig. 2). For each population, we performed feature selection, data integration, and subclustering independently to utilize features relevant for distinguishing cell subtypes^43–45^ (Fig. 2).

CD4^+^ T cells comprised 13 functional subtypes and one unclassified CD4^+^ T, including Th1, Th2, Th17, regulatory T cells, and tissue-resident memory cells (Fig. 2a–c). Analysis of immune cell subpopulations revealed several significant differences between POAG patients and healthy controls. The CD4^+^ T-cell compartment demonstrated profound alterations in subset distribution and activation states. The data revealed significant expansion in POAG patients of regulatory T cells (Tregs) (*P* = 3.64 × 10^−17^), Th2 cells (*P* = 1.61 × 10^−9^), and cytotoxic CD4^+^ T cells (*P* = 4.40 × 10^−12^), along with increased proportions of resting memory CD4^+^ T cells (*P* = 3.00 × 10^−12^), effector memory CD4^+^ T cells (*P* = 4.71 × 10^−7^), and T follicular helper (Tfh) cells (*P* = 1.84 × 10^−3^). Conversely, naive CD4^+^ T cells (*P* = 4.74 × 10^−7^) and Th1 (*P* = 7.89 × 10^−19^) were markedly reduced in POAG patients. These findings reveal a complex immunological landscape characterized by enhanced regulatory and memory T-cell responses coupled with diminished naive and Th1-cell populations. The reduction in naive CD4^+^ T cells, combined with increased memory subsets, suggests accelerated T-cell differentiation and enhanced antigen-experienced T-cell responses in POAG pathogenesis.

CD8^+^ T cells were divided into naive, effector, and exhausted populations, with γδT cells and MAIT cells forming distinct clusters (Fig. 2d–f). The CD8^+^ T-cell compartment exhibited profound alterations in differentiation and activation states. The data revealed significant expansion of CD8^+^ naive T cells (*P* = 6.69 × 10^−5^) and CD8_transitional T cells (*P* = 0.0032), suggesting enhanced T-cell priming in POAG patients. Conversely, the proportions of CD8^+^ effector GZMK T cells were dramatically reduced (*P* = 3.30 × 10^−16^), whereas the proportions of CD8^+^ exhausted T cells were decreased (*P* = 1.2 × 10^−3^), indicating diminished cytotoxic effector function. Notably, γδT cells were significantly elevated (*P* = 1.54 × 10^−2^), potentially reflecting enhanced innate-like T-cell responses. The natural killer cells were separated into CD56^bright^, CD56^dim^ and CD56^intermediate^ subsets (Fig. 2g–i). The total percentage of NK cells was significantly lower in POAG patients than in controls, as mentioned above, yet NK cell subtype analysis revealed no significant differences in the CD56^bright^ or CD56^dim^ populations. This pattern suggests a global reduction in NK cell numbers rather than subtype-specific alterations in POAG pathogenesis.

B lymphocytes in PBMCs were resolved into multiple transcriptionally distinct subpopulations, including naive, memory, double-negative (DN), plasmablast, and transitional B-cell subsets, as visualized by UMAP embedding (Fig. 2j, k, i). Compared with those in controls, quantitative comparisons revealed significant alterations in the composition of B-cell subsets in POAG patients (Fig. 2i). Notably, the proportion of total memory B cells was significantly decreased in POAG patients (*P* = 2.52 × 10^−4^), as was the proportion of double-negative (DN) B cells (*P* = 0.0426). In contrast, POAG patients presented significant increases in the proportions of GC-derived memory B cells (*P* = 8.35 × 10^−8^), atypical memory B cells (*P* = 2.05 × 10^−4^), and autoreactive DN B cells (*P* = 0.0143) relative to controls. These findings indicate a selective depletion of conventional memory and DN B cells, accompanied by an expansion of GC-derived, atypical, and autoreactive B-cell subsets in POAG. This altered B-cell compartment composition suggests a shift in B-cell differentiation and activation states, which may contribute to the immunological dysregulation observed in POAG. Consistent with these cellular alterations, multiple studies have reported increased levels of autoreactive antibodies, including antibodies against HSP27, HSP60, α-fodrin, GFAP, and other neuronal or metabolic antigens, in the serum, aqueous humor, and tears of POAG patients.^46–48^ These autoantibodies have been implicated in RGC loss, mitochondrial dysfunction, and disease progression, suggesting a role for B-cell-mediated autoimmunity in POAG pathogenesis.

The myeloid subsets of PBMCs were delineated into eight classes, including classical monocytes, nonclassical/intermediate monocytes, and other myeloid populations (Fig. 2m–o). The myeloid compartment revealed substantial remodeling, characterized by marked expansion of intermediate monocytes (*P* = 1.19 × 10^−8^), classical monocytes (*P* = 1.31 × 10^−2^), and macrophage-like monocytes (*P* = 8.04 × 10^−9^), alongside increased moDCs (*P* = 4.53 × 10^−3^). In contrast, cDC1s (*P* = 3.54 × 10^−5^) and pDC plasmacytoids (*P* = 5.0 × 10^−4^) were significantly reduced. These changes suggest enhanced proinflammatory myeloid responses with diminished tolerogenic DC populations. Collectively, these findings reveal a complex immunological signature in POAG characterized by enhanced T-cell memory formation and regulatory responses coupled with diminished cytotoxic effector function and naive T-cell populations. The observed shifts in immune cell composition, including increased myeloid cell activation, suggest that comprehensive immune system remodeling may contribute to POAG pathogenesis through enhanced antigen presentation, modified T-cell differentiation trajectories, and altered inflammatory cytokine profiles.^49,50^

### Extensive and cell type-specific remodeling of signaling pathways in circulating immune cells from POAG patients

**Fig. 3:**
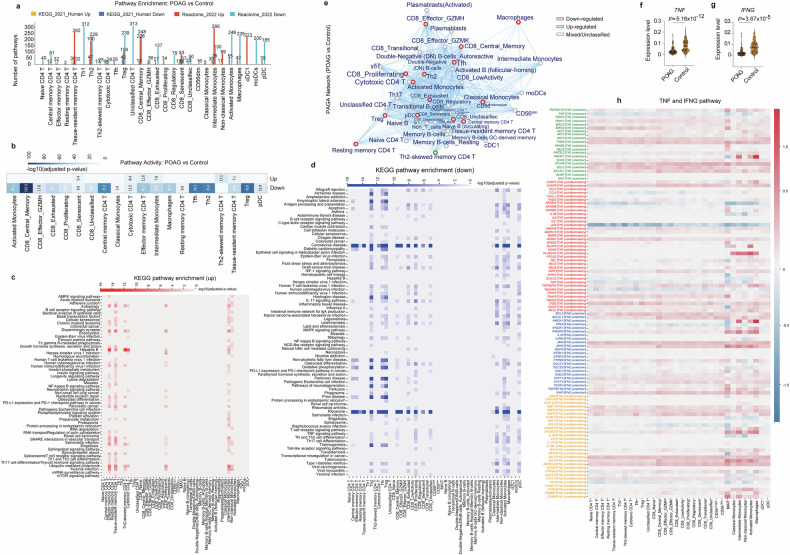
Extensive and cell type-specific pathway dysregulation in circulating immune cells from POAG patients. **a** Bar plot showing the number of significantly upregulated (red) and downregulated (blue) KEGG and Reactome pathways in each immune cell subtype between POAG patients and controls. **b** Heatmap summarizing the overall pathway activity score (−log10 adjusted *P* value, up- or down-) for each major immune cell subtype in POAG patients versus controls by Partition-based graph abstraction (PAGA) analysis. **c** Heatmap of significantly upregulated KEGG pathways (rows) across immune cell subtypes (columns) in POAG patients versus controls. The color intensity reflects the significance and direction of change. **d** Heatmap of significantly downregulated KEGG pathways (rows) across immune cell subtypes (columns) in POAG patients versus controls. **e** PAGA network showing the relationships and pathway activity changes among immune cell subtypes in POAG patients versus controls. Node color indicates up- (red) or downregulation (green) of pathway activity. **f**, **g** Violin plots showing the relative expression levels of the *TNF* (**f**) and *IFNG* (**g**) genes in PBMCs from POAG patients and controls. *P* values are indicated. **h** Combined pathway heatmap (natural style) displaying the relative activity of selected immune, metabolic, and neurodegenerative pathways (rows, grouped and color-coded by pathway type) across immune cell subtypes (columns) in POAG patients versus controls. The color scale represents normalized pathway activity scores

To clarify the functional impact of transcriptional changes in POAG immune cells, we performed pathway enrichment analysis via KEGG and Reactome via GSEApy. Across 30 of 54 immune cell subtypes, we identified widespread pathway alterations (*P*_adjusted_ < 0.05; Fig. 3a, Supplementary Table 5). The reactome consistently identified more altered pathways than did the KEGG, reflecting its broader coverage. The number and direction of altered pathways varied considerably among different cell types, highlighting highly heterogeneous, cell type-specific pathway remodeling in POAG. Across 29 distinct immune cell subtypes, we identified widespread downregulation of pathway activity, with the most significant reductions observed in CD8_Central_Memory cells (−log10(adj_pval) = 102.0), Treg cells (79.8), Tfh cells (76.3), Th2 cells (73.4), and central memory CD4 T cells (71.3). Other notably downregulated populations included activated monocytes (47.1), effector memory CD4^+^ T cells (45.7), and macrophages (41.4) (Fig. 3b). These results demonstrate complex immune reprogramming in POAG, with some populations exhibiting both activation and suppression, whereas others are dominated by pathway downregulation.

### Convergence of immune, metabolic, and neurodegenerative signaling in POAG

Pathway enrichment analysis revealed a striking convergence of immune, metabolic, and neurodegenerative processes in POAG patients (Fig. 3c, d, Supplementary Table 5, Supplementary Fig. 1). The most significant pathway alterations were observed in CD8_Central_Memory cells, with ribosome biogenesis (*P*_adjusted_ = 9.18 × 10^−103^) showing extreme downregulation. Treg cells exhibited marked suppression of ribosomal pathways (*P*_adjusted_ = 1.48 × 10^−80^), whereas Tfh cells presented similar patterns (ribosome: *P*_adjusted_ = 5.04 × 10^−77^). Th2 cells demonstrated the most extensive suppression of neurodegenerative pathways, including Parkinson’s disease (*P*_adjusted_ = 1.66 × 10^−19^), Alzheimer’s disease (*P*_adjusted_ = 1.63 × 10^−10^⁻), Huntington’s disease (*P*_adjusted_ = 2.20 × 10^−11^), and oxidative phosphorylation (*P*_adjusted_ = 2.81 × 10^−17^). Notably, POAG immune cells exhibited dual dysregulation: widespread downregulation of ribosomal and mitochondrial pathways coexisting with selective upregulation of neuroprotective signaling in specific subsets (Fig. 3c). Neurotrophin signaling was upregulated in tissue-resident memory CD4^+^ T cells (*P*_adjusted_ = 1.59 × 10^−3^), whereas ubiquitin-mediated proteolysis was increased in effector memory CD4^+^ T cells (*P*_adjusted_ = 1.43 × 10^−8^) and tissue-resident memory CD4^+^ T cells (*P*_adjusted_ = 8.66 × 10^−8^). Autophagy pathways were upregulated in effector memory CD4^+^ T cells (*P*_adjusted_ = 4.34 × 10^−6^) and tissue-resident memory CD4^+^ T cells (*P*_adjusted_ = 7.97 × 10^−6^) (Fig. 3c), suggesting compensatory mechanisms for cellular stress and neuroprotection.

Partition-based graph abstraction (PAGA) network analysis^51^ revealed comprehensive immune cell remodeling characterized by widespread downregulation across multiple key immune cell types (Fig. 3e). The network revealed distinct clusters of B-cell and plasmablast populations, T-cell subsets, and myeloid/monocyte/DC populations, with intricate connectivity patterns reflecting developmental trajectories and functional relationships. Notably, the PAGA network showed predominant downregulation (red nodes) across multiple critical immune cell types, including various activated B cells, Tregs, resting memory CD4^+^ T cells, macrophages, classical monocytes, and pDCs. This widespread suppression aligns with our pathway analysis findings and suggests a fundamental alteration in immune cell homeostasis in POAG. Conversely, Th2-skewed memory CD4^+^ T cells were specifically upregulated (green node), indicating the selective enhancement of this particular T-cell subset.

Collectively, these findings demonstrate that POAG is characterized by broad suppression of translational, metabolic, and neurodegenerative pathways, alongside selective activation of neurotrophic and immune signaling in distinct immune subsets, reflecting systemic suppression of protein synthesis.^52,53^ This dual landscape suggests that immune cells in POAG not only participate in immune surveillance and inflammation but also may directly influence neurodegenerative processes through altered metabolic and neurotrophic signaling. These insights highlight the complex immune–neural crosstalk in POAG and suggest potential therapeutic targets aimed at restoring immune cell homeostasis and neuroprotection.

### Cell type-specific analysis reveals dual-polarized IFNG and TNF signaling in POAG PBMCs

Both TNF-α and IFN-γ are well recognized for their dual roles in the immune system, acting as mediators of neuroprotection as well as potent drivers of inflammation.^54,55^ Both TNF and IFNG expression levels were significantly lower in PBMCs from POAG patients than in those from controls (*TNF: P* = 5.16 × 10^−12^ *IFNG: P* = 3.67 × 10^−5^ Fig. 3f, g), findings validated in an independent cohort (Supplementary Fig. 2). To dissect immune dysregulation in POAG, we performed cell type-specific analyses of IFNG and TNF signaling across all 54 PBMC subtypes (Fig. 3h). Despite this global suppression, detailed analysis revealed a dual-polarized transcriptional landscape (Fig. 3h). Inflammatory signaling components downstream of *IFNG*, such as *JAK1/2*, *MAPK14*, and *IRF1*, are upregulated in specific cell subsets. Moreover, the expression of neuroprotective and antiapoptotic genes (*BCL2*, *BCL2L1*, *XIAP*, *SOD2*, and *HMOX1*) was upregulated, particularly in regulatory and memory T cells and myeloid populations. TNF signaling showed a similar duality: while TNF itself was downregulated, proinflammatory mediators (*CASP1/8, RIPK1/3, TRAF2/5, TAB2*) were upregulated in T cells and myeloid cells, and neuroprotective factors (*BCL2, BCL2L1, CFLAR, BIRC2/3, AKT1/2/3, PTEN, HMOX1*) were elevated in regulatory and memory T cells and monocytes. These results reveal that POAG PBMCs exhibit compartmentalized transcriptional reprogramming, with concurrent suppression of core cytokines and selective activation of both inflammatory and neuroprotective pathways in distinct immune subsets. This dual-polarized signaling landscape may underlie the complex immune adaptation and altered neuroimmune homeostasis observed in POAG.

### Cellular composition and gene expression analysis in POAG patients with high vs normal IOP

**Fig. 4:**
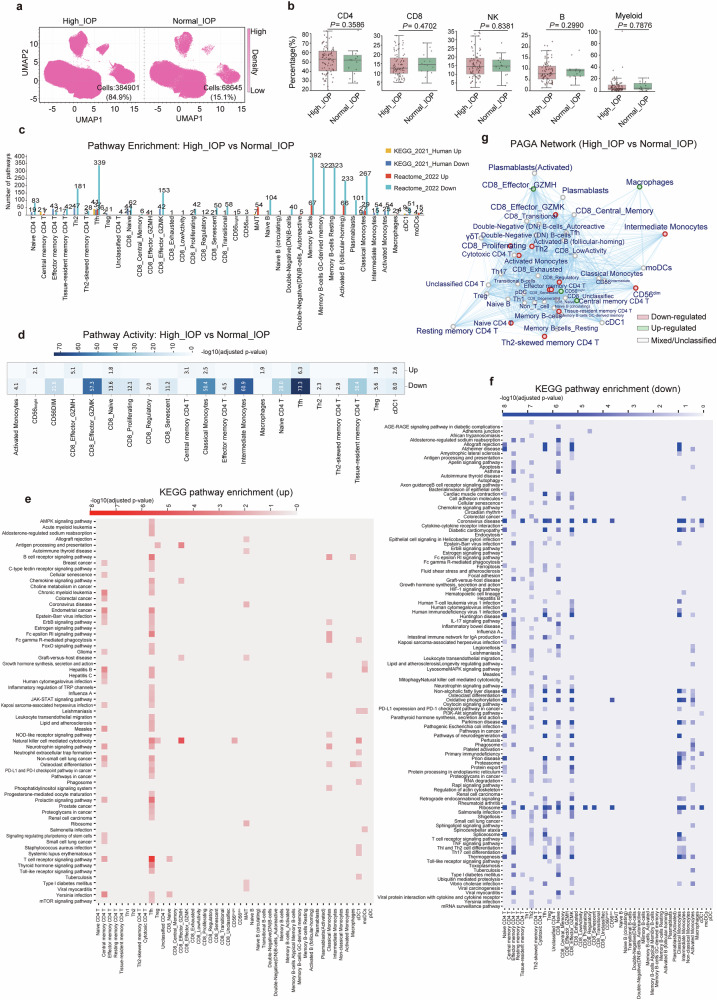
Comparative analysis of immune cell composition and pathway dysregulation between high-IOP and normal-IOP POAG patients. **a** UMAP plots showing the distribution of major immune cell populations in POAG patients with high intraocular pressure (IOP) and normal IOP. **b** Box plots comparing the proportions of major immune cell types (CD4^+^ T cells, CD8^+^ T cells, NK cells, B cells, and myeloid cells) between high-IOP and normal-IOP POAG patients. *P* values are indicated for each comparison. **c** Bar plot showing the number of significantly upregulated (red) and downregulated (blue) pathways (KEGG and Reactome) in each immune cell subtype when high-IOP and normal-IOP POAG patients were compared. **d** Heatmap summarizing the overall pathway activity score (−log10 adjusted *P* value, up- or down-) for each major immune cell subtype in high-IOP patients versus normal-IOP POAG patients by PAGA analysis. **e** Heatmap of significantly upregulated KEGG pathways (rows) across immune cell subtypes (columns) in high-IOP versus normal-IOP POAG patients. The color intensity reflects the significance and direction of change. **f** Heatmap of significantly downregulated KEGG pathways (rows) across immune cell subtypes (columns) in high-IOP versus normal-IOP POAG patients. **g** PAGA network showing the relationships and pathway activity changes among immune cell subtypes in high-IOP versus normal-IOP POAG patients. Node color indicates up- (red) or downregulation (green) of pathway activity

Comparative analysis of PBMC composition and transcriptomes between POAG patients with elevated versus normal IOP revealed distinct disease mechanisms. Among 98 POAG patients, 83 (84.7%) presented with elevated IOP, whereas 15 (15.3%) maintained normal IOP levels (Fig. 4a). Analysis of major PBMC types revealed no statistically significant compositional differences between the high-IOP and normal-IOP groups (Fig. 4b, Supplementary Fig. 3), suggesting that overt shifts in the cellular proportions are not a primary feature distinguishing these subgroups. However, the imbalanced sample sizes between groups (normal IOP: *n* = 15 vs high IOP: *n* = 83) may have limited the statistical power to detect subtle cellular differences.

Despite this limitation, KEGG and Reactome pathway analysis of the DEGs revealed profound functional distinctions, with 341 significantly upregulated and 758 significantly downregulated pathways identified across 38 of 54 immune cell subtypes (Fig. 4c, Supplementary Table 5). PAGA pathway activity analysis revealed widespread downregulation in high-IOP patients, with the most pronounced reductions observed in Tfh cells (activity score: −73.3, −log10(adj_pval) >70), intermediate monocytes (−60.9), CD8^+^ effector GZMK cells (−57.3), and classical monocytes (−50.4) (Fig. 4d).

Further detailed KEGG pathway analysis revealed distinct patterns of cellular dysfunction in POAG patients stratified by IOP status (Fig. 4e, f). The high-IOP group exhibited significantly more severe pathway suppression compared to the normal-IOP group, with Tfh cells showing the most profound dysregulation in the high-IOP subgroup. Among the 15 pathways showing extreme significance in Tfh cells from high-IOP patients, ribosome biogenesis (*P*_adjusted_ = 4.93 × 10^−74^) and oxidative phosphorylation (*P*_adjusted_ = 1.28 × 10^−29^) were the most prominently affected. Intermediate monocytes and CD8^+^ effector GZMK cells also presented profound pathway suppression in the high-IOP group, with ribosome pathways downregulated at *P*_adjusted_ = 1.30 × 10^−61^ and *P*_adjusted_ = 5.15 × 10^−58^, respectively, while showing less severe suppression in the normal-IOP group. Strikingly, the high-IOP group showed selective upregulation of immune activation pathways that were not observed in the normal-IOP group, including T-cell receptor signaling in Tfh cells (*P*_adjusted_ = 5.03 × 10^−7^) and NK cell-mediated cytotoxicity in CD8⁺ effector GZMH⁺ cells (*P*_adjusted_ = 7.41 × 10^−6^). Despite these IOP-specific differences, convergent dysregulation patterns were observed in both high-IOP and normal-IOP groups, including widespread downregulation of ribosomal pathways (e.g., activated B cells: case vs control, *P*_adjusted_ = 1.70 × 10^−104^) and pathways associated with neurodegenerative diseases across multiple cell types. Neurodegenerative pathways, including Parkinson disease, Alzheimer’s disease, and Huntington’s disease, were consistently downregulated across multiple immune cell populations in both IOP groups. These findings collectively demonstrate that elevated IOP in POAG patients is associated with significantly more severe suppression of fundamental cellular processes, particularly protein synthesis, energy metabolism, and neurodegenerative disease-related pathways, while maintaining selective activation of cytotoxic immune responses that are not observed in normal-IOP patients.

PAGA network analysis revealed distinct cellular landscape alterations in high-IOP patients (Fig. 4g). Among the 54 immune cell subtypes, 10 populations, including CD8^+^ effector GZMK cells, Th2 cells, CD8^+^ proliferating cells, cytotoxic CD4^+^ T cells, intermediate monocytes, CD8^+^ regulatory cells, CD56dim NK cells, naive CD4^+^ T cells, memory B cells, and Th2-skewed memory CD4^+^ T cells, were significantly downregulated. Conversely, 6 populations were upregulated: CD8^+^ effector GZMH cells, macrophage-like cells, CD56^bright^ NK cells, central memory CD4^+^ T cells, naive B cells (circulating), and tissue-resident memory CD4^+^ T cells. The remaining 28 populations displayed mixed or unclassified expression patterns. This cellular remodeling indicates the suppression of regulatory and memory cell populations while maintaining cytotoxic effector functions, particularly in CD8^+^ T cells and NK cells, in high-IOP POAG patients.

The identification of this IOP-specific immunometabolic signature, characterized by suppressed protein synthesis/neurodegeneration-associated pathways coupled with enhanced cytotoxic immunity, provides crucial mechanistic insights into the relationship between IOP status and immune cell dysfunction in POAG. The enhanced cytotoxic responses observed in high-IOP patients may represent adaptive, yet potentially detrimental, immune mechanisms. Modulating these IOP-specific pathways presents promising targets for personalized neuroprotective therapeutic strategies aimed at high-IOP POAG, potentially preserving immune surveillance while mitigating glaucomatous damage.

### Systemic gene expression signatures robustly classify POAG status

**Fig. 5:**
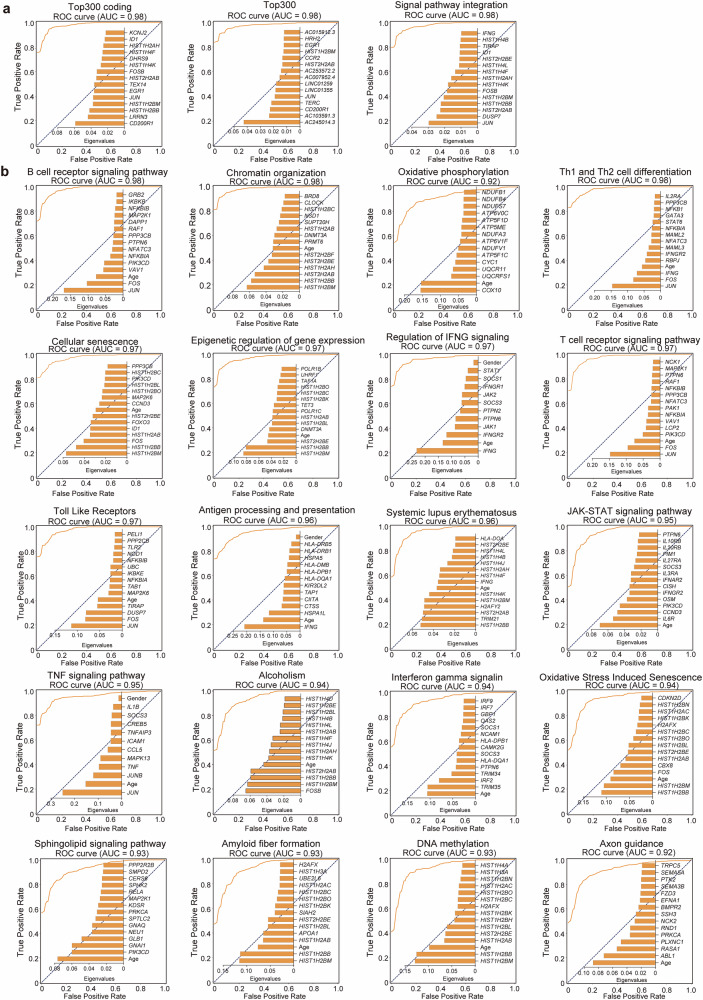
Machine learning-based prediction of POAG using gene expression signatures. **a** Receiver operating characteristic (ROC) curves for three random forest models distinguishing POAG patients from controls: the top 300 coding DEGs, the top 300 overall DEGs, and an integrated model based on the top differentially expressed signaling pathways. **b** ROC curves for individual pathway-based models with areas under the curve (AUCs) > 0.90, with insets showing the top 15 most important gene features for each pathway. For all the models, the data were randomly split into training (49%), validation (21%), and test (30%) sets with balanced class distributions

Random forest classifiers were trained using features derived from the top DEGs or genes within the 25 most dysregulated signaling pathways, including age and sex as covariates. Models utilizing either the top 300 DEGs or the integrated pathway gene set achieved exceptional classification accuracy, yielding near-perfect discrimination (AUC = 0.98; Fig. 5a), highlighting the profound systemic transcriptional changes in POAG. Further analysis revealed strong predictive power inherent in individual biological pathways (Fig. 5b, Supplementary Table 6). Notably, 20 pathways yielded models with AUCs ≥0.90. Pathways central to the identified immune dysregulation, including B-cell receptor signaling (AUC = 0.98), TNF signaling (AUC = 0.95), and interferon gamma signaling (AUC = 0.93), were the top performers.

To address potential concerns about overfitting and ensure the robustness of our findings, we conducted independent validation via quantitative polymerase chain reaction (qPCR) on a separate cohort of 59 samples (31 cases and 28 controls). This independent validation provides evidence that our molecular signatures represent genuine biological differences between POAG patients and controls rather than artifacts of overfitting. This validation study confirmed the exceptional predictive performance, with the TNF signaling pathway achieving an AUC = 0.91 and the regulation of the IFNG signaling pathway achieving an AUC = 0.78, and identified key predictive features, including *JUN*, *STAT1*, T*NF*, and *IFNGR1*, as the most important discriminators between POAG patients and controls (Supplementary Fig. 4).

The robust classification performance, particularly driven by genes within the TNF, IFNG, and B-cell receptor signaling pathways, strongly links these systemic molecular signatures to POAG status. Our independent qPCR validation study provides compelling evidence that these molecular signatures are not artifacts of overfitting but represent genuine biological differences between POAG patients and controls. The identification of key predictive features, including *JUN* (importance value: 0.25) and *TNF* (importance value: 0.13), offers valuable insights into the molecular mechanisms underlying POAG pathogenesis (Supplementary Fig. 3). These findings underscore the biological significance of the observed immune pathway dysregulation, especially the systemic dampening of TNF and IFNG signaling, in POAG and suggest potential therapeutic targets for future interventions.

### Cell type-specific eQTL analysis reveals genetic control of immune dysregulation in POAG

**Fig. 6:**
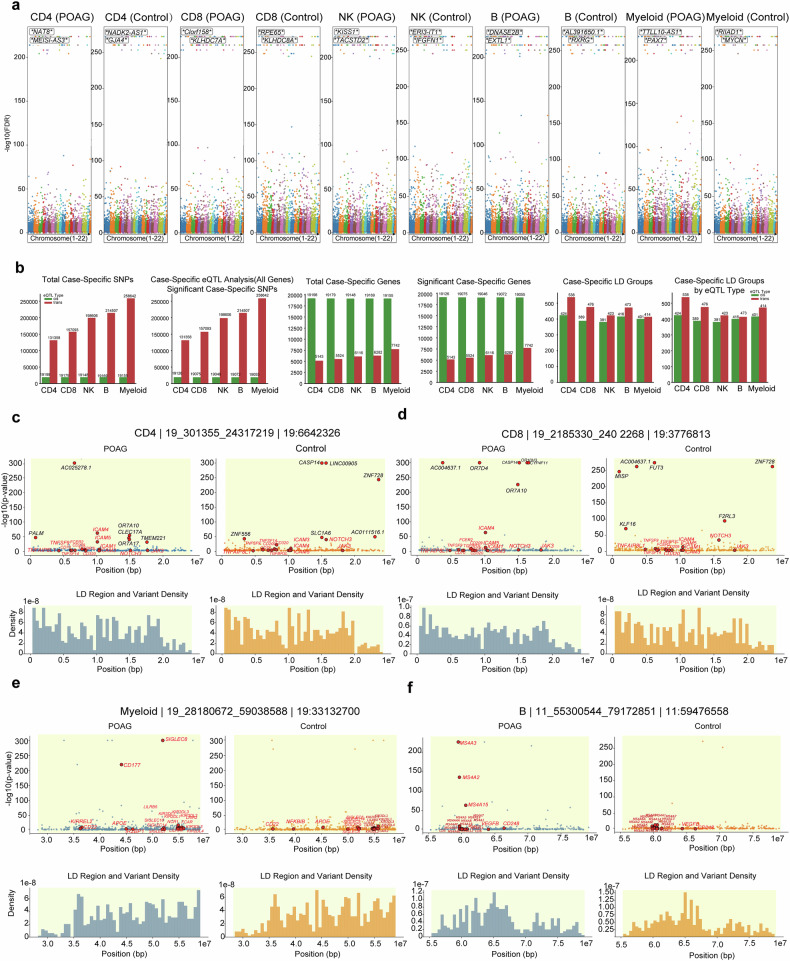
Cell type-specific eQTL mapping reveals context-dependent genetic regulation of immune cells in POAG. **a** Manhattan plots of cis-eQTL associations (–log₁₀[FDR]) for five major PBMC populations (CD4⁺ T, CD8⁺ T, NK, B, and myeloid cells) in POAG patients and controls. The top disease- or control-specific loci and genes are highlighted. **b** Quantitative summary of uniquely detected eQTLs across cell types in POAG patients. Bar plots show the total number of uniquely detected SNPs in POAG patients, significant SNP–gene pairs, regulated genes, and LD groups, stratified by cis- and trans-eQTLs. Trans-eQTLs constitute the majority of uniquely detected regulatory events in POAG patients, with myeloid and CD4⁺ T cells exhibiting the highest counts. **c**–**f** Representative locus-level analyses of CD4⁺ T cells (**c**), CD8⁺ T cells (**d**), myeloid cells (**e**), and B cells (**f**) in POAG and control samples. For each locus, the upper panel shows the –log₁₀[P] values for SNP‒gene associations, the middle panel displays the LD region and variant density. POAG-specific eQTL signatures are observed at key immune loci, including *ICAM4, NOTCH3, JAK3, CD177, SIGLEC8*, the *MS4A* gene cluster, and *VEGFB*, with regulatory architectures distinct from those of controls

Building upon our discovery of extensive immune cell remodeling and pathway dysregulation in POAG PBMCs, we integrated genome-wide genotyping data with our single-cell transcriptomic profiles to map cell type-specific eQTLs across five major PBMC populations. Following quality control and imputation procedures, we analyzed 20,844,451 SNPs for cis- and trans-eQTL associations via Tensortl,^56–58^ adjusting for demographic factors and principal components (Fig. 6, Supplementary Figs. 5 and 6, Dataset 1).

#### Divergent eQTL signatures despite overall conservation

Cis-eQTL analysis revealed 19,247 significant SNP‒gene pairs across all PBMC populations, with strikingly similar numbers between POAG patients and controls (99.4–99.8% concordance; Fig. 6a, b; Supplementary Fig. 5). While the global cis*-*eQTL landscape appeared largely conserved, a subset of cis-eQTLs was uniquely detected in cases or controls. In stark contrast, trans-eQTL analysis revealed profound differences between POAG patients and controls across all cell types (Supplementary Fig. 5). POAG patients presented markedly reduced trans-eQTL activity, with the most pronounced reductions in CD8^+^ T cells (71% reduction: 157,119 vs 547,157 trans-eQTLs) and NK cells (65% reduction: 198,811 vs 572,945 trans-eQTLs). Similar patterns were observed across other cell populations: B cells (44% reduction), CD4^+^ T cells (66% reduction), and myeloid cells (32% reduction). This dramatic reduction in trans-eQTL activity provides a genetic basis for the extensive immune cell remodeling we documented in POAG. The profound reduction in CD8^+^ T-cell and NK cell trans-eQTL activity directly correlates with our observation of diminished CD8^+^ T-cell (*P* = 2.53 × 10^−7^) and NK cell (*P* = 2.19 × 10^−5^) populations in POAG patients. This genetic evidence supports our hypothesis that POAG is characterized by a disruption of cytotoxic immune cell function, potentially contributing to altered immune surveillance and neuroprotective mechanisms (Supplementary Fig. 5). This dramatic reduction in trans-eQTL activity provides genetic evidence for the extensive immune cell remodeling we documented in POAG. The reduction in trans-eQTL activity across all cell types suggests a global alteration in long-range genetic regulation in POAG, potentially reflecting disease-associated changes in transcriptional networks and regulatory architecture. This finding aligns with our pathway analysis revealing widespread suppression of key immune activation pathways, including TNF and IFNG signaling, which we demonstrated to be robust classifiers of POAG status.

#### Uniquely detected eQTLs in POAG patients reveal unique genetic regulatory signatures

Analysis of uniquely detected eQTLs in POAG patients revealed distinct genetic regulatory signatures in POAG, with trans-eQTLs constituting the majority across all major immune cell types. Substantial numbers of uniquely detected trans-eQTLs in POAG patients were identified in B cells (214,507), CD4^+^ T cells (131,358), CD8^+^ T cells (157,093), myeloid cells (258,642), and NK cells (198,606) (Fig. 6b, Dataset 2). The high prevalence of uniquely detected trans-eQTLs in myeloid cells in POAG patients coincided with increased myeloid cell proportions (*P* = 0.033) and activation states observed in POAG patients, suggesting a potential link between genetic regulation and the proinflammatory myeloid responses characteristic of the disease. Similarly, the extensive uniquely detected trans-eQTL activity in CD4^+^ T cells in POAG patients paralleled the expansion of this population and regulatory T-cell subsets in POAG patients. While these associations point to a possible contribution of trans-acting genetic variants to immune cell remodeling in POAG, further functional studies are needed to establish direct causal relationships between these regulatory variants and disease-associated immune phenotypes.

#### Large LD blocks orchestrate coordinated regulatory networks

To further dissect the genetic regulatory architecture underlying immune dysregulation in POAG, we performed fine-mapping of large LD eQTLs within representative genomic regions across major PBMC subsets. Regional association plots revealed striking differences in the eQTL landscape between POAG patients and controls. At the chromosome 19 locus, both CD4^+^ and CD8^+^ T cells from POAG patients presented a broader and more intense spectrum of significant eQTL associations, with prominent regulatory peaks at immune-related genes such as *TNFAIP8, ICAM1, ICAM5*, and *NOTCH3*, whereas controls presented distinct regulatory signals at alternative loci, including *CASP14, ZNF728*, and *LINC00955* (Fig. 6c, d). Similarly, in the myeloid compartment, POAG samples presented strong eQTL signals for genes such as *SIGLEC9, CD177*, and *APOE*, whereas controls presented a divergent regulatory profile (Fig. 6e). In B cells, the POAG-specific eQTL landscape was dominated by immunoglobulin gene clusters (e.g., *MS4A3, MS4A4E*, and *MS4A6A*), in contrast to the control group, which presented regulatory peaks in alternative immune genes (Fig. 6f). These findings highlight disease- and cell type-specific divergence in the genetic regulation of key immune loci, supporting a model in which POAG is associated with context-dependent remodeling of regulatory networks that orchestrate immune cell function.

To further dissect the genetic regulatory architecture underlying immune dysregulation in POAG, we performed fine-mapping of eQTLs within the IFNG and TNF genomic regions across major PBMC subsets (Supplementary Fig. 7a–j). Fine-mapping of the IFNG and TNF loci across major PBMC subsets revealed marked disease- and cell type-specific divergence in genetic regulation. Compared with those in controls, the IFNG region in POAG patients presented a broader and more pronounced spectrum of significant eQTLs in CD4^+^ T cells, CD8^+^ T cells, NK cells, B cells, and myeloid cells, with increased density and effect size of regulatory associations (Supplementary Fig. 7a–e). Similarly, the TNF locus in POAG showed an expanded and intensified eQTL landscape, particularly in CD4^+^ T cells and myeloid cells, with multiple variants exerting strong effects on TNF and neighboring cytokine genes, in contrast to the sparse signals observed in controls (Supplementary Fig. 7f–j). These findings indicate that POAG is associated with context-dependent remodeling of eQTL architecture at key cytokine loci, potentially driving immune dysregulation in disease.

#### Integration with POAG GWAS loci reveals cell type-specific regulatory patterns

Integration of single-cell eQTL data with established POAG GWAS loci revealed pronounced cell type-specific regulatory architectures (Supplementary Fig. 8a, Supplementary Table 7). Cell type-specific eQTL mapping revealed distinct regulatory patterns; for example, synaptic function genes such as *PCLO* had the strongest regulatory effects on CD4⁺ T cells (FDR = 5.47 × 10^−24^). Caveolin-mediated signaling genes, exemplified by *CAV2*, were strongly associated with CD8⁺ T cells (FDR = 3.07 × 10^−32^). In NK cells, transcriptional regulatory genes such as *SIX1* were most significantly regulated (FDR = 1.54 × 10^−214^), whereas B cells presented strong eQTL signals for stress response genes, including *CRHR1* (FDR <1e-300). Myeloid cells were associated with eQTLs for Wnt signaling genes, such as *CAV2* (FDR = 3.43 × 10^−11^). Collectively, these results highlight the highly cell type-specific nature of genetic regulation in POAG, emphasizing the importance of the immune context in mediating the functional impact of GWAS risk loci (Supplementary Table 7).

Quantitative summary analyses (Supplementary Fig. 8a) demonstrated that both the number of significant SNPs and the number of regulated genes varied substantially by cell type, with myeloid cells and CD4^+^ T cells exhibiting the highest counts of significant eQTLs and LD groups. Detailed locus-level analyses further highlighted disease- and cell type-specific regulatory divergence. For example, at a representative B-cell locus on chromosome 11 (Supplementary Fig. 8b), POAG cases presented strong eQTL signals for *SPDYC*, *AP003774.1*, and *SNX15*, whereas controls presented distinct regulatory peaks for *ZNHIT2*, *BATF2*, and *EFEMP2*. Similarly, at an NK cell locus on chromosome 22 (Supplementary Fig. 8c), POAG-specific eQTLs were observed for *SSTR3* and *TEX33*, whereas control samples presented regulatory associations with *CDC42EP1*, *LGALS2*, and *KCNJ4*. These findings underscore the context-dependent nature of genetic regulation at POAG risk loci, with distinct sets of target genes and regulatory variants in disease versus control states. Collectively, these results demonstrate that the integration of GWAS and single-cell eQTL data reveals highly cell type- and disease-specific regulatory patterns at POAG risk loci, providing mechanistic insight into how genetic variation may drive immune cell dysfunction in POAG.

#### Genetic–immune axis provides a mechanistic framework

These regulatory hotspots align with our single-cell transcriptomic findings of systemic immune cell remodeling in POAG, characterized by increased CD4^+^ T-cell and myeloid cell proportions alongside diminished CD8^+^ T and NK cell populations. The integration of eQTL analysis with our single-cell transcriptomic findings reveals a comprehensive picture of POAG pathogenesis, where genetic variants may exert their effects through long-range regulatory mechanisms that coordinate immune cell function and differentiation. This genetic–immune axis provides a potential mechanistic framework for understanding how systemic immune alterations may contribute to retinal neurodegeneration in POAG, suggesting a link between genetic predisposition and local pathology.

### SMR analysis implicates causal effects of gene expression in specific immune cells on POAG risk

To elucidate the causal relationships between genetic variation, immune cell gene regulation, and POAG susceptibility, we performed SMR analyses integrating our cell type-resolved eQTL data with published POAG GWAS summary statistics.^59^ This approach enables the identification of loci where genetic variants influence POAG risk through modulation of gene expression in specific immune cell subsets.

**Fig. 7:**
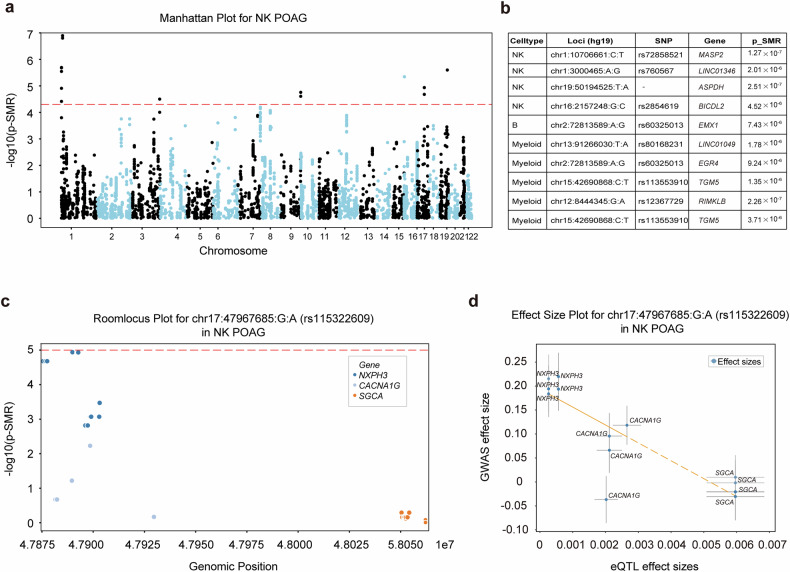
SMR analysis reveals that cell type-specific genetic regulation of gene expression is associated with POAG risk. **a** Manhattan plot of genome-wide summary data-based Mendelian randomization (SMR) results for POAG in NK cells. Each point represents a gene–SNP pair; the horizontal red dashed line indicates the Bonferroni-corrected significance threshold (*P*_*_SMR*_ = 2.85 × 10^−6^). **b** Supplementary Table 8 summarizing the most significant SMR associations (*P*_*_SMR*_ < 1 × 10^−5^) across immune cell types, including cell type, genomic locus, SNP, target gene, and *P*_*_SMR*_ value. **c** Regional association (locus) plot for rs115322609 in NK cells, showing SMR *P* values for genes in the region (*NXPH3, CACNA1G, SGCA*). The red dashed line marks the significance threshold. **d** Effect size plot for rs115322609 in NK cells, comparing eQTL effect sizes (x-axis) and GWAS effect sizes (y-axis) for *NXPH3, CACNA1G*, and *SGCA*. The error bars indicate confidence intervals

Across the 17,516 SNP‒gene pairs tested, we applied a Bonferroni-corrected significance threshold (*P* < 2.85 × 10^−6^) to define genome-wide significance (Fig. 7a, b; Supplementary Fig. 9; Supplementary Table 8). Our analysis revealed several loci with either genome-wide or suggestive evidence of colocalization between eQTL and POAG GWAS signals in NK cells, B cells, and myeloid populations (Fig. 7b). Notably, in NK cells, we identified significant associations for *MASP2* (rs72858521, *P*_*_SMR*_ = 1.27 × 10^−7^), *LINC01346* (rs760567, *P*_*_SMR*_ = 2.01 × 10^−6^), and *ASPDH* (chr19:50194525:T:A, *P*_*_SMR*_ = 2.51 × 10^−6^), among others. These findings suggest that genetic regulation of these genes in NK cells may contribute to POAG pathogenesis, which is consistent with our observation of reduced NK cell abundance and cytotoxic function in patients. In B cells and myeloid cells, additional loci, such as *EMX1, LINC01049*, and *EGR4*, also demonstrated suggestive colocalization, highlighting the diversity of immune cell types through which genetic risk may be mediated.

To further elucidate these relationships, we examined the locus at chr17:47967685:G:A (rs115322609) in NK cells (Fig. 7c, d). Regional association and effect size analyses revealed that the expression of *NXPH3* at this locus had a concordant positive effect on both the eQTL and GWAS datasets, suggesting that increased *NXPH3* expression may increase POAG risk. In contrast, *SGCA* exhibited a negative GWAS effect size despite a positive eQTL effect, indicating a potentially protective role for increased *SGCA* expression. These results underscore the complexity of genetic regulation, where a single variant may exert pleiotropic effects on multiple genes with distinct functional consequences.

Importantly, while SMR analysis provides statistical evidence for colocalization and putative causality, it cannot fully exclude confounding factors due to linkage disequilibrium or horizontal pleiotropy. Thus, these findings should be interpreted as supporting, rather than definitive, evidence for causal mediation. Collectively, our SMR and colocalization analyses revealed that POAG-associated genetic variants may exert their effects through cell type-specific regulation of gene expression in peripheral immune cells. These results provide a mechanistic framework linking genetic risk to immune cell remodeling in POAG and identify specific genes and cell types for future functional investigations.

### Functional validation: impaired TNF and IFNG expression affects retinal vulnerability to glaucomatous injury

To establish the baseline TNF response to injury, we characterized the temporal expression of the Tnfa gene and TNFα protein in both retinas and PBMCs following LPS/NMDA treatment in WT mice (Supplementary Fig. 10). Both compartments exhibited rapid and significant upregulation of *Tnf* mRNA and protein, peaking at 12–48 h (*P* < 0.001), followed by a decline and a late-phase rebound at 7 days (Supplementary Fig. 10a, b). This temporal analysis provides insights into the dynamic nature of TNF signaling in POAG and helps explain the apparent discrepancy between our PBMC findings and existing ocular TNF literature. Notably, compared with 0 h, LPS/NMDA stimulation led to a significant increase in the levels of CD4^+^ and CD8^+^ T-cell markers in the retina at 48 h (Supplementary Fig. 10c).

**Fig. 8:**
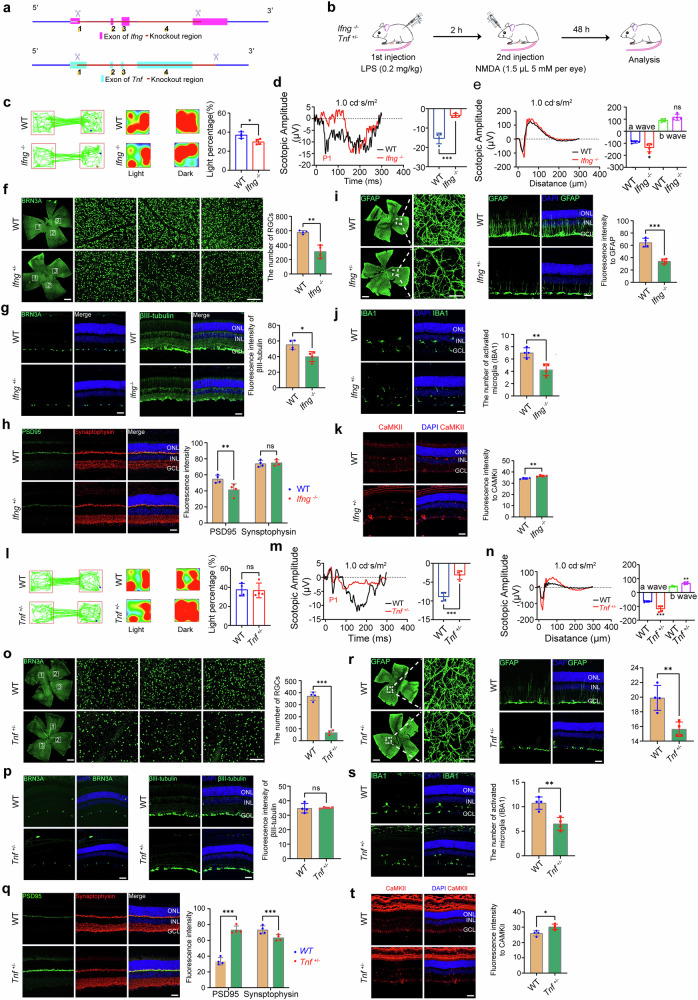
Genetic deficiency in *Ifng* or *Tnf* exacerbates retinal neurodegeneration and visual dysfunction in an LPS/NMDA-induced injury model. **a** Targeting strategy for generating *Ifng*^*-/-*^ and *Tnf* ^+/-^ mice. **b** Experimental timeline: Mice received intraperitoneal LPS (0.2 mg/kg) followed 2 h later by intravitreal NMDA (1.5 µL, 5 mM); analyses were performed 48 h post-NMDA. **c** Light‒dark box test (LDBT) showing reduced time spent in the light compartment by *Ifng*^-/-^ mice compared with WT controls (*n* = 4 WT, *Ifng*^-/-^ per group). **d** Representative visual evoked potential (VEP) waveforms and quantification of N1‒P1 amplitudes showing attenuation in *Ifng*^-/-^ mice (*n* = 4 WT, *Ifng*^-/-^ per group). **e** Representative scotopic electroretinogram (ERG) waveforms and quantification of a-wave and b-wave amplitudes showing a reduction in *Ifng*^-/-^ mice (*n* = 4 WT, *Ifng*^-/-^ per group). **f** cross immune cell typeBrn3a (RGC marker) and quantification showing reduced RGC density in Ifng⁻/⁻ mice (*n* = 4 WT, *Ifng*^-/-^ per group). Scale bar: 20 µm Scale bar: 50 µm. **g**–**k** Representative retinal cryosections and quantification of fluorescence intensity for **g** βIII-tubulin (neuronal marker) and Brn3a (RGCs in the GCL), **h** PSD95 and Synaptophysin (synaptic markers in the IPL), **i** representative retinal whole mounts stained for GFAP (astrocyte marker) and immunofluorescence and fluorescence intensity quantification of GFAP in *Ifng*^-/-^ mice (*n* = 4 WT, *Ifng*^-/-^ per group). Scale bar: 20 µm; (**j**) IBA1 (a microglial marker) and **k** CaMKII (an activity marker) in WT and *Ifng*^-/-^ mice (*n* = 4 WT, *Ifng*^-/-^ per group). Scale bars: 20 µm. **l** LDBT showing reduced time in light for the *Tnf* ^+/-^ mice compared with the WT mice (*n* = 4 WT, *Tnf* ^+/-^ per group). **m** Representative visual evoked potential (VEP) waveforms and quantification of N1‒P1 amplitudes showing attenuation in *Tnf*^-/-^ mice (*n* = 4 WT, *Tnf*^-/-^ per group). **n** ERG analysis showing reduced amplitudes in *Tnf* ^+/-^ mice (*n* = 4 WT, *Tnf* ^+/-^ per group). **o** RGC quantification from Brn3a-stained whole mounts showing loss in *Tnf* ^+/-^ mice (*n* = 4 WT, *Tnf* ^+/-^ per group). Scale bar: 20 µm; scale bar: 50 µm. **p**–**t** Representative retinal cryosections and quantification of fluorescence intensity: **p** βIII-tubulin (neuronal marker) and Brn3a (RGCs in the GCL), **q** PSD95 and Synaptophysin (synaptic markers in the IPL), **r** representative retinal whole mounts stained for GFAP (astrocyte marker) and immunofluorescence and fluorescence intensity quantification of GFAP in *Ifng*^-/-^ mice (*n* = 4 WT, *Ifng*^-/-^ per group). Scale bar: 20 µm Scale bar: 50 µm; **s** IBA1 (microglia marker), **t** CaMKII (activity marker) in WT vs *Tnf* ^+/-^ mice (*n* = 4 WT, *Tnf* ^+/-^ per group). Scale bars: 20 µm. The data are presented as the means ± SEMs. Statistical significance was determined by unpaired Student’s t test (*P* < 0.05, *P* < 0.01, *P* < 0.001). GCL ganglion cell layer, IPL inner plexiform layer

Our human PBMC analysis revealed consistent downregulation of *TNF* and *IFNG* in POAG patients (Fig. 3f, g). To functionally validate this systemic‒local axis, we employed genetic mouse models with targeted disruption of the *TNF* or *IFNG* sexpression. Wild-type (WT) mice were compared with Ifng knockout (*Ifng*^*-/-*^) and Tnf heterozygous (*Tnf*^*+/-*^) mice, revealing the diminished signaling capacity observed in human POAG patients (Fig. 8a, b). Western blot analysis confirmed the complete absence of IFN-γ in *Ifng*^*-/-*^ mice and reduced TNFα in *Tnf*^*+/-*^ mice (Supplementary Fig. 10d, f). Genetic ablation studies revealed that, compared with WT control mice, both *Ifng*^*-/-*^ and *Tnf*^*+/-*^ mice presented significant reductions in CD4^+^ T-cell populations (*P* < 0.001 and *P* < 0.01, respectively), whereas *Tnf*^*+/-*^ mice also presented decreased CD8^+^ T-cell expression (*P* < 0.01) (Supplementary Fig. 10e, g). Flow cytometry analysis of the PBMC composition revealed distinct alterations in the genetically modified mice. *Ifng*^*-/-*^ mice presented significantly reduced percentages of CD161^+^ cells (WT: 6.35% vs *Ifng*^*-/-*^: 4.99%, *P* < 0.01) (Supplementary Fig. 11), which parallels the reduced NK cell populations observed in human POAG patients. Conversely, *Tnf*^*+/-*^ mice presented a decrease in FoxP3^+^ regulatory T cells (WT: 94.70% vs. *Tnf*^*+/-*^: 78.50%, *P* < 0.01) and an increase in CD161^+^ cells (WT: 3.00% vs. *Tnf*^*+/-*^: 4.46%, *P* < 0.01) (Supplementary Fig. 12), showing patterns that differ from the human POAG phenotype. The mouse models show distinct immune cell alterations, with the *Ifng*^*-/-*^ model providing direct functional validation of our human PBMC findings, while the *Tnf*^*+/-*^ model reveals complex TNFα-dependent immune regulation that may involve additional factors in human POAG. These findings establish IFN-γ as a critical regulator of immune cell composition, with its deficiency resulting in specific alterations that parallel key features of the human POAG immune phenotype.

#### Retinal functional outcomes in genetically modified mice

Ifng deficiency profoundly worsened retinal outcomes following injury. Compared with WT control mice, *Ifng*^*-/-*^ mice presented exacerbated visual deficits (light‒dark box: *P* < 0.05; Fig. 8c), severe central visual pathway dysfunction (VEP amplitudes: *P* < 0.001; Fig. 8d), and significantly greater RGC loss (Brn3a^+^ cells: *P* < 0.01; Fig. 8f, g). Scotopic ERG analysis revealed an increased magnitude of the a wave (*P* < 0.01; Fig. 8e), whereas synaptic integrity was compromised with decreased PSD95 immunoreactivity (*P* < 0.01; Fig. 8h). The reactive gliosis response was blunted, with significantly lower GFAP (*P* < 0.001; Fig. 8i) and IBA1 signals (*P* < 0.01; Fig. 8j), whereas CaMKII showed increased staining intensity (*P* < 0.01; Fig. 8k), but P-CaMKII decreased (*P* < 0.05; Supplementary Fig. 10e).

Similarly, in *Tnf*^*+/-*^ mice, the retina was significantly more vulnerable to injury compared to WT. While light–dark box behavior was unchanged (ns; Fig. 8l), visual function was severely impaired (VEP amplitudes: *P* < 0.001; Fig. 8m), and *Tnf*^*+/-*^ animals presented increased magnitudes of both scotopic ERG a- and b-wave amplitudes (*P* < 0.01 for both; Fig. 8n). Histologically, *Tnf*^*+/-*^ mice presented significantly greater RGC loss (*P* < 0.001; Fig. 8o, p), with divergent synaptic changes: increased PSD95 immunoreactivity (*P* < 0.001; Fig. 8q) but decreased synaptophysin expression (*P* < 0.001; Fig. 8q). The glial response was also blunted (GFAP: *P* < 0.01; Fig. 8r; IBA1: *P* < 0.01; Fig. 8s), while CaMKII staining was increased (*P* < 0.05; Fig. 8t), but P-CaMKII expression was decreased (*P* < 0.05; Supplementary Fig. 10g).

#### Mitochondrial dynamics are disrupted in the absence of IFNG or TNF signaling following retinal injury

To further elucidate the mechanisms underlying increased neuronal vulnerability, we examined mitochondrial dynamics in the injured retina via both Western blot and immunofluorescence analyses. Compared with wild-type control mice, both *Ifng*^*-/-*^ and *Tnf*^*+/-*^ mice presented a significant reduction in OPA1 expression and increased levels of DRP1 and FIS1, indicating a shift toward enhanced mitochondrial fission and impaired fusion (Supplementary Fig. 13). Quantitative analysis via immunofluorescence staining confirmed these changes across the ganglion cell layer (GCL), inner nuclear layer (INL), and outer nuclear layer (ONL) (Supplementary Fig. 13).

#### Integration of findings and interpretation of paradoxical responses

Collectively, these findings demonstrate that genetic attenuation of TNF or IFNG signaling renders the retina more susceptible to injury, resulting in exacerbated neurodegeneration, impaired visual function, and maladaptive molecular responses (Fig. 8c–t). The paradoxical increase in ERG amplitudes and CaMKII levels, despite worsened neurodegeneration, likely reflects maladaptive hyperexcitability or stress responses rather than neuroprotection. Elevated CaMKII, in the context of RGC loss and VEP decline, represents a marker of calcium dysregulation and excitotoxicity rather than functional enhancement. These results support a model in which balanced TNF and IFNG signaling is essential for retinal resilience, and their deficiency disrupts the delicate equilibrium between inflammatory and neuroprotective mechanisms, as observed in POAG.

## Discussion

POAG pathogenesis involves a complex interplay of factors leading to RGC death, with current therapies targeting IOP offering incomplete protection.^1,2,60^ The contribution of the immune system, particularly systemic immunity, has been suspected but poorly defined.^4,61–63^ Here, we provide a comprehensive, high-resolution dissection of the circulating immune system in POAG by integrating large-scale human scRNA-seq, genome-wide genetics, and functional validation in mouse models. Our study reveals a distinct systemic immune signature in POAG, characterized by altered cell composition and, most strikingly, a sophisticated dual-transcriptional landscape in which inflammatory and neuroprotective signaling pathways coexist and interact.

Our findings reveal that POAG is not simply a disorder of compartmentalized immune dysregulation but rather a complex immunometabolic syndrome characterized by a dysregulated balance between inflammatory and neuroprotective signaling. Notably, we identified a dual-transcriptional landscape in circulating immune cells in which both the inflammatory and neuroprotective pathways are simultaneously engaged. This duality is particularly evident in the regulation of TNF and IFNG signaling, which play both proinflammatory and neuroprotective roles. The context-dependent nature of these cytokines likely explains why simple genetic ablation models do not fully recapitulate the human disease state. Our single-cell atlas revealed marked remodeling of the PBMC compartment in POAG, with a reduction in NK and CD8^+^ T cells and an expansion of CD4^+^ T cells—including regulatory T cells (Tregs). This shift away from cytotoxic potential is consistent with prior flow cytometry studies,^23,24^ but our work provides unprecedented transcriptomic and functional detail. Importantly, we observed a sophisticated bifurcation of TNF and IFNG pathway gene expression across immune lineages, with the upregulation of inflammatory mediators (e.g., *JAK1/2, MAPK14, CASP1/8*, and *RIPK1/3*) in some populations and robust neuroprotective gene expression (e.g., *BCL2, BCL2L1, XIAP, HMOX1*, and *SOD2*) in others. This dual-transcriptional signature suggests that the immune system in POAG is not simply overactivated or suppressed but is engaged in a maladaptive attempt to balance neuroinflammatory stress with compensatory neuroprotection.

These transcriptional changes are mirrored by significant alterations in immune cell composition. Enhanced inflammatory signaling in expanded myeloid compartments, together with the upregulation of neuroprotective genes in regulatory and memory T-cell populations, points to a complex, context-dependent immune response. The robust neuroprotective signature, particularly in myeloid cells and Tregs, may reflect endogenous mechanisms attempting to counterbalance chronic inflammatory stress in POAG patients. Integration of genetic data adds a critical mechanistic layer. We show that a substantial fraction of established POAG GWAS risk loci act as cis-eQTLs in specific immune cell types, providing a cellular context for genetic susceptibility. SMR analysis further prioritized potentially causal relationships, implicating genes such as *MASP2, EMX1*, and *NXPH3* in NK or B cells as mediators of POAG risk. These findings strongly suggest that inherited genetic risk for POAG is, at least in part, mediated through altered peripheral immune function.

Reconciling our findings with the literature on TNF expression in POAG requires consideration of compartmentalized immune responses. Our findings suggest a compartmentalized TNF response in POAG, where systemic TNF-α expression in PBMCs is downregulated, whereas local ocular TNF-α expression may remain elevated. This pattern could reflect a complex immunoregulatory mechanism in which systemic immune cells downregulate TNF production in response to chronic local inflammation in the eye. This compartmentalized response helps explain the apparent contradiction between our PBMC findings and existing findings showing elevated TNF in ocular and plasma contexts.

A central advance of our study is the functional validation of these dysregulated pathways. While PBMC profiling offers a powerful window into systemic immune states, establishing their relevance to CNS pathology is essential. Our demonstration that genetic deficiency of *Ifng* or *Tnf* in mice exacerbates retinal neurodegeneration and visual dysfunction following injury provides compelling evidence that the dual-transcriptional landscape observed in human POAG represents a maladaptive, rather than protective, state.

The paradoxical molecular responses we observed—including increased ERG amplitudes and elevated CaMKII levels despite worsened neurodegeneration—suggest that TNF-α and IFNγ deficiency disrupts the delicate equilibrium between inflammatory activation and neuroprotective mechanisms rather than simply removing pathogenic factors. The divergent glial responses observed in *Tnf*^+/-^ mice (enhanced astrogliosis, reduced microglia) further highlight the pleiotropic and context-dependent roles of TNF in modulating neuroinflammation and glial reactivity.^64^ Our findings also help reconcile apparent contradictions in the TNF literature. We suggest a compartmentalized TNF response in POAG, where systemic TNF-α expression in PBMCs is downregulated, whereas local ocular TNF-α expression may remain elevated. This pattern could reflect a complex immunoregulatory mechanism in which systemic immune cells downregulate TNF-α production in response to chronic local inflammation in the eye, explaining the discrepancy between our PBMC findings and existing reports of elevated TNF-α in ocular and plasma contexts.

Our study has several limitations. PBMC transcriptomes, while robust classifiers of disease status (AUC = 0.98), serve as sentinel indicators of systemic immune status and may not fully capture the complexity of the ocular immune environment. Establishing direct causality between peripheral immune changes and RGC death in human POAG remains challenging. The acute LPS/NMDA injury model, although informative, does not fully recapitulate the chronic nature of human POAG. Importantly, our IOP subanalysis (Fig. 4) has limited statistical power because of the imbalanced sample size in the normal IOP group (*n* = 15) compared with the high IOP group (*n* = 83). This imbalance restricts our ability to draw definitive conclusions about IOP-independent mechanisms in POAG pathogenesis, and these findings should be interpreted with caution and considered exploratory in nature. All the human subjects were of Chinese Han ancestry, which may limit their applicability to other populations; future studies should include diverse cohorts and direct replication. While our integration with global multiethnic GWAS loci provides preliminary evidence for broader relevance, direct replication studies in diverse populations are essential to validate the cross-population applicability of our findings. Longitudinal studies tracking immune changes with disease progression, investigations of immune cell trafficking to the eye, and more sophisticated mouse models that recapitulate the dual-transcriptional landscape of human POAG will be essential for further mechanistic insight.

Our findings also highlight the broader value of PBMC profiling in neurodegenerative disease research. Prior studies have identified microRNA and transcriptomic biomarkers in PBMCs for Alzheimer’s disease and Parkinson’s disease,^65^ underscoring the utility of peripheral immune signatures in understanding CNS pathology. By comparing the scRNA-seq features of POAG with those of other neurodegenerative and related diseases, it is clear that each condition possesses unique transcriptomic hallmarks. However, the dual inflammatory–neuroprotective landscape we describe in POAG is, to our knowledge, a novel finding with potential implications for immune–neural interactions in other neurodegenerative contexts.

In summary, our integrated multiomics and functional approach reveals a link between systemic immune dysregulation and retinal vulnerability in glaucoma patients. We provide a cellular and molecular atlas of circulating immunity in POAG, link genetic risk to immune cell function, and demonstrate that disruption of TNF/IFNG signaling homeostasis is associated with increased retinal vulnerability in a relevant disease model. These findings suggest that therapeutic strategies should target compartmentalized dysregulation rather than broad suppression of TNF/IFNG pathways. Our findings identify distinct immune cell subtypes with cell type-specific transcriptional signatures, providing a foundation for investigating precision immunotherapies in POAG patients. Future studies may investigate single-cell-guided approaches to selectively modulate neuroprotective pathways in regulatory populations while restoring effector cell function, potentially representing a shift from broad immunosuppression to context-specific immune modulation in glaucoma therapeutics. Our work provides mechanistic insights and a resource for POAG research.

## Materials and methods

### Sample collection and ethics approval

Informed consent was obtained from all participants, comprising 110 primary open-angle glaucoma (POAG) patients and 110 healthy controls from the Sichuan Provincial People’s Hospital. All the human subjects in this study were of Chinese Han ancestry, which may limit the generalizability of our findings to other populations. The study was approved by the Ethics Committee of Sichuan Provincial People’s Hospital (approval no. 2019 (036)). All participants were of Chinese Han ancestry and had no history of COVID-19 infection. Peripheral blood mononuclear cells (PBMCs) were isolated, Ficoll-separated, and cryopreserved by the Sichuan Provincial Key Laboratory for Human Disease Gene Study. Demographic details, including sex, age, and BMI, are provided in Supplementary Table 1.

### Study design and inclusion criteria

This study included three cohorts of POAG patients with high intraocular pressure (IOP) or normal IOP (NPG) for scRNA-sequencing and TNF expression validation. Extensive ophthalmic examinations were conducted, excluding participants with autoimmune, infectious, neurodegenerative, or metabolic diseases. The inclusion criterion for POAG patients with high IOP was an open anterior chamber angle. Samples were collected at the time of initial POAG diagnosis. Controls were required to have Chinese Han ancestry, age ≥40 years, and no history of glaucoma.

### PBMC processing and sequencing

PBMCs were processed using CEDARLANE’s Lympholyte® Cell Separation media. The cells were isolated and washed, and dead cells were removed before being loaded onto a 10X Chromium Controller. Libraries were prepared via the 10X Chromium Next GEM Single Cell 3ʹ Reagent Kit v3.1 and sequenced on a NovaSeq 6000. Data processing was performed via Cell Ranger version 3.0.2.

### Single-cell RNA-seq data analysis

A total of 1,416,923 cell-containing droplets were sequenced. Data preprocessing involved normalization, PCA, and Louvain clustering via Scanpy. Cell annotation was performed with typical markers. Batch effects were corrected with sample ID, sex, age, and BMI as covariates. Differential expression and pathway analyses were conducted via GSEA.

### Random forest classifier predictions

A random forest classifier was constructed using scikit-learn to analyze the gene expression data. The dataset was split into training, validation, and test sets. The feature importance was assessed, and the model performance was evaluated via the F1 score, ROC curve, and AUC value.

### Genotyping and imputation

All the samples were genotyped via the Axiom hNCG SNP array. After quality control, 295,132 SNPs were used for imputation on the CHN100K server via Minimac4,^64^ resulting in 20,844,451 SNPs for cis-eQTL analysis.

### eQTL and SMR analysis

eQTL analysis was performed via tensorQTL,^56^ which incorporates the top PCs, age, sex, and BMI as covariates. SMR analysis^59^ integrates eQTL and GWAS data^66^ to identify genetic variants influencing gene expression and traits.

### Real-time quantitative PCR

RNA was extracted from PBMCs. cDNA was synthesized from single-cell sequencing samples. RT‒qPCR was conducted using specific primers for TNF-α and Actin. Relative expression was calculated via the 2-ΔΔCt method, with statistical significance assessed via Student’s t test.

### Animal model and retinal injury

All animal experiments were approved by the Sichuan Academy of Medical Sciences and Sichuan Provincial People’s Hospital. Eight- to ten-week-old wild-type (WT) C57BL/6J, *Ifng*^*-/-*^, and *Tnf*^*+/-*^ mice received an intraperitoneal injection of LPS (0.2 mg/kg), followed by an intravitreal injection of NMDA (1.5 µl of 5 mM) under anesthesia 2 h later. Analyses were performed 48 h post-NMDA injection.

### Immunofluorescence and microscopy

Retinal ganglion cells (RGCs) were visualized and quantified via whole-mount immunofluorescence staining for Brn3a (Abcam, ab245230). Retinal cryosections (12 µm) were stained for various markers, including Brn3a, GFAP (CST, #80788), IBA1 (ABclonal, A19776), PSD95 (ABclonal, A0131), Synaptophysin (Proteintech, 17785-1-AP), CaMKII (Invitrogen, PA5-19128), and βIII-tubulin (ABclonal, A17913). The sections were counterstained with DAPI and imaged using a Zeiss LSM900 confocal microscope.

### Visual function assessment

Scotopic flash electroretinograms (ERGs) and visual evoked potentials (VEPs) were recorded from dark-adapted, anesthetized mice via a Celeris system (Diagnosys Ltd.) to assess retinal function and visual pathway integrity, respectively.

### Behavioral testing

The light‒dark box test was used to assess anxiety-like behavior and photophobia. The mice were allowed to explore the apparatus for 5 min, and the time spent in each compartment and transition were recorded via automated tracking software (Shanghai Xinruan).

### Flow cytometry

Peripheral blood immune cell populations were analyzed via multiparameter flow cytometry in heparinized whole blood. Antibodies against CD3, CD4, CD8a, CD19, CD161/NK1.1, CD25, and FOXP3 were used for surface and intracellular staining (following fixation/permeabilization for FOXP3). Intracellular IFN-γ production by T cells was assessed following 4–6 h of ex vivo stimulation with PMA/ionomycin and brefeldin A/monensin. The data were acquired on a flow cytometer and analyzed via FlowJo software.

### Statistical analysis

The data were analyzed via unpaired Student’s t tests or one-way ANOVA with appropriate post hoc tests (GraphPad Prism). *P* < 0.05 was considered statistically significant. The data are presented as the means ± SEMs unless otherwise stated.

The detailed step-by-step protocols are available in the Supplementary Methods.

## Supplementary information

Supplementary information

Supplementary information

Supplementary information

Supplementary information

## Data Availability

Processed clean h5ad scRNA-seq files (samples_clean_h5ad.7z) are available through https://zenodo.org (10.5281/zenodo.17114800). Genotype data can be obtained by contacting the corresponding author (huangluling@yeah.net).
