# Supplementary material for: Single-cell RNA-seq reveals cell type-specific molecular and genetic associations with primary open-angle glaucoma
Source: Signal Transduct Target Ther. 2025 Oct 10;10:338. doi: 10.1038/s41392-025-02438-x (PMC12511369; doi:10.1038/s41392-025-02438-x)
Supplement: Supplementary file 1 — Supplementary information [file 41392_2025_2438_MOESM1_ESM.docx]

Supplementary Materials for

**Single−cell RNA-seq reveals cell type-specific molecular and genetic associations to primary open-angle glaucoma**

Huaping Tian^1,#^, Yuhong Chen^2,#^, Tujing Zhao^1,#^, Lin Ye^1,#^, Hongjing Li^1^, Zheng Li^1^, Wenqiao Qiu^1^,Wentao Wang^2^, Runze Li^1^, Fulin Liu^1^, Haojue Xue^3^, Ruilin Liao^1^, Chao Qu^3^, Jie Li^3^, Liang Zou^4^, Yi Shi^1^, Zhenglin Yang^1,5,^*  and Lulin Huang^1,^*

*Corresponding authors. Email: [huangluling@yeah.net](mailto:huangluling@yeah.net) (Lulin Huang) or [yangzhenglin@cashq.ac.cn](mailto:yangzhenglin@cashq.ac.cn) (Zhenglin Yang)

**This PDF file includes:**

Materials and Methods

Supplementary Figures 1-13

**Materials and Methods**

Sample collection

Informed consent was obtained from all samples sequenced in this study. 110 POAG patients and 110 healthy controls were collected from the Sichuan Provincial People's Hospital. These studies were approved by the Ethics Committee of Sichuan Provincial People’s Hospital (approval no. 2019 (036)). None of the participants had ever been infected with COVID-19. Peripheral blood mononuclear cells were isolated from donors, Ficoll separated, and cryopreserved by the Sichuan Provincial Key Laboratory for Human Disease Gene Study. The gender, age, BMI, and information are listed in Table S1.

Study Subject Enrollment and Diagnosis with POAG:

This study involved three cohorts of primary open-angle glaucoma (POAG) patients with high intraocular pressure (IOP) or normal IOP (NPG) from the Han Chinese population. These cohorts were used for a scRNA-sequencing study (scRNA-seq) and the replication cohort was used for TNF expression validation.

All participants underwent extensive ophthalmic examinations by ophthalmologists following a standardized protocol. Participants with autoimmune, infectious, neurodegenerative, or metabolic diseases were excluded. Samples were collected at the time of initial POAG diagnosis.

Inclusion Criteria for POAG Cases with High IOP (HPG):

1. Anterior chamber angle open.

2. Adult-onset POAG: age of disease onset (or diagnosis) of ≥20 years.

3. Axial length of ≤27.0 mm in both eyes.

4. Visual field with any one eye with characteristic glaucomatous damage (on reliable visual fields) consistent with nerve fiber layer loss. If a single eye is affected, it should be the one with increased IOP, and the other eye should at least have an enlarged vertical cup-disc ratio (≥0.6) or elevated IOP (≥22 mm Hg) if there is no visual field defect.

5. Lack of any other eye diseases that could severely affect visual acuity or the visual field, such as retinitis pigmentosa, proliferative diabetic retinopathy (PDR), age-related macular degeneration with choroidal neovascularization (AMD-CNV), or pathological myopia.

6. Lack of any secondary glaucoma or cause for elevated IOP, such as Posner Schlossman syndrome, pigment dispersion syndrome, exfoliation syndrome, steroid-induced glaucoma, neovascular glaucoma, uveitic glaucoma or angle recession glaucoma.

7. Absence as a member of a pedigree in which glaucoma was inherited as a Mendelian trait (affecting three successive generations with three cases in total).

Inclusion Criteria for POAG Cases with Normal IOP (NPG):

Same as for HPG, except with normal IOP (<21 mm Hg).

Inclusion Criteria for Controls:

1. Chinese Han ancestry.

2. Age at enrollment of ≥40 years.

3. IOP of ≤21 mm Hg.

4. A vertical cup-disc ratio of ≤0.5.

5. Absence of very narrow angle.

6. Axial length between 21.0 and 27.0 mm in both eyes.

7. No history of glaucoma or elevated IOP.

8. No eye diseases affecting visual acuity or visual field.

9. No family history of glaucoma.

Participants who self-reported having autoimmune, infectious, neurodegenerative, or metabolic diseases during their health check-up were excluded from the study.

PBMC processing, RNA and antibody-derived tags (ADT) library preparation, and

sequencing

CEDARLANE’s Lympholyte® Cell Separation density gradient centrifugation media (Lot#25-127) has been specifically designed for the isolation of viable Frozen PBMC lymphocytes. Briefly, each of the 220 blood samples was diluted with an equal volume of 1X PBS (pH7.4) added into a 15 ml tube. Gently layer the cell suspension or whole blood over top of the Lympholyte® making sure not to mix the two layers and centrifuged at 800 × g for 20 min at room temperature. After centrifugation, carefully remove the cells at the interface using a pasteur pipette and transfer them into a new centrifuge tube for washing. Enriched cells were washed with PBS and centrifuged at 800 × g for 10 min twice. Dead cells were removed using a Dead Cell Removal Kit (Miltenyi, Lot#130-090-101) Cells were loaded onto a 10X Chromium Controller. Cell numbers and viability were measured using a Countess II Automated Cell Counter (Thermo Fisher Scientific). Cells were loaded onto a 10X Chromium Controller. Following library preparation using the published 10X Chromium Next GEM Single Cell 3ʹ Reagent Kits v3.1 Solution protocol, libraries were sequenced on the NovaSeq6000 at a depth of more than 2,000 reads/cell. Analysis pipelines in Cell Ranger version 3.0.2 were used for sequencing data processing. Transcriptome data were processed by running Cell Ranger count with transcriptome = refdata-cellranger-GRCh38-1.2.0.

Single-cell RNA-seq data preprocessing

A total of 1,416,923 cell-containing droplets were obtained after sequencing from 220 samples.The analysis of single-cell RNA sequencing (scRNA-seq) data involves several key steps to ensure high-quality results. This workflow utilizes Scanpy, a popular Python library for single-cell analysis, and CellHint for cell annotation and batch effect correction. We used 10x Genomics to profile peripheral blood mononuclear cells (PBMCs) from 220 independent samples obtained from the Sichuan Provincial People's Hospital, including 110 POAG patients and 110 healthy controls passed QC, all of Chinese Han ancestry (Supplementary Table 1). After quality control and doublet removal using Scrublet, red blood cells (RBCs) with 200 > UMI > 10,000 genes and cells lost typical PBMC markers were removed; the final dataset contained a total of 903,377 high-quality cells and 36,598 genes.

*Data Preprocessing and Quality Control*:

Loading Data: Begin by loading the scRNA-seq data into an AnnData object using Scanpy. This object efficiently stores the expression matrix and associated metadata.

Normalization: Normalize the data to account for differences in sequencing depth across cells, using total-count normalization followed by log transformation.

*Dimensionality Reduction and Clustering:*

PCA and Nearest Neighbors: Perform principal component analysis (PCA) to reduce dimensionality, followed by computing a neighborhood graph of cells.

Clustering: Louvain clustering analysis of normalized and batch-corrected single-cell transcriptomic profiles suggested no observable batch effects on the distribution of cells between POAG and controls.

*Cell type identification and subclustering:*

For fine-grained cell type annotation, we performed iterative subclustering of major PBMC lineages (B cells, CD4^+^ T cells, CD8^+^ T cells, monocytes, and NK cells). For each lineage, single-cell transcriptomes were subsetted and reanalyzed using Scanpy (v1.9.1). Dimensionality reduction was performed using principal component analysis (PCA), with batch correction by Harmony, regressing out sample ID, sex, and age to minimize technical and biological confounding effects. Neighborhood graphs were constructed using the top 30 principal components and 15 nearest neighbors, followed by Leiden clustering (resolution = 3.0). UMAP was used for visualization.

Cluster identities were assigned based on the expression of canonical marker genes curated for each lineage. Marker gene sets included pan-immune and lineage-specific markers, as well as functional and disease-relevant genes. For each cluster, marker gene expression was visualized using dot plots, and cluster-specific expression matrices were exported for downstream analysis. Cell type assignments were further refined by integrating marker expression patterns with unsupervised clustering results. Highly expressed genes in each cell population were also used as supplementary evidence for cell type identification.

To comprehensively characterize the cellular landscape of PBMCs, we performed high-resolution single-cell RNA sequencing followed by unsupervised clustering and dimensionality reduction. After rigorous quality control and normalization, we applied the Louvain algorithm to identify transcriptionally distinct clusters, which were visualized using Uniform Manifold Approximation and Projection (UMAP). Cell type annotation was achieved by integrating canonical marker gene expression profiles with established single-cell PBMC atlases and recent literature.

CD4⁺ T cell subtypes were distinguished by the expression of *CD3D* and *CD4*, and further resolved into naive, memory, effector, regulatory, and specialized helper subsets based on the combinatorial expression of key markers such as *LEF1, CCR7, IL7R, CD44, FOXP3, TBX21, GATA3, RORC*, and *CXCR5*. This approach enabled the identification of naive, central memory, effector memory, resting memory, activated/migratory, tissue-resident, and regulatory T cells, as well as Th1, Th2, Th17, and T follicular helper (Tfh) populations.

CD8⁺ T cell subtypes were defined by the presence of *CD3D* and CD8A/*CD8B*, and further classified into naive, central memory, effector, exhausted, proliferating, regulatory, and senescent populations. Subtype delineation was based on the expression of markers such as *LEF1, CCR7, SELL, GZMA, GZMB, PRF1, TOX, MKI67*, and *FOXP3*, as well as the identification of mucosal-associated invariant T (MAIT) and γδT cell subsets through the detection of T*RAV1-2, SLC4A10, TRDC*, and *TRGC* gene expression.

Natural killer (NK) cell subtypes were annotated by the expression of *NCAM1* (CD56) and *FCGR3A* (CD16), and further stratified into CD56^bright^, CD56^dim^, and CD56^intermediate^ populations. These subsets were characterized by distinct expression patterns of cytotoxicity-related genes, including *GZMA, GZMB, PRF1, GNLY,* and *NKG7*, as well as maturation and activation markers.

B cell subtypes were identified by the expression of *CD19, CD79A*, and *MS4A1*, and further resolved into naive, memory, double-negative (DN), plasmablast, and transitional populations. Subclassification was based on the expression of *LEF1, TCL1A, SELL, CD27, BANK1, FCRL3, CXCR5, TBX21, FCRL5, CD38, SDC1, JCHAIN*, and *PRDM1*, allowing for the discrimination of GC-derived, atypical, and autoreactive memory B cells, as well as plasmablasts and transitional B cells.

Myeloid cell subtypes were annotated by the expression of *CD14* and *FCGR3A*, and further divided into classical, intermediate, and non-classical monocytes, macrophages, plasmacytoid and conventional dendritic cells, monocyte-derived dendritic cells, and activated monocytes. These populations were distinguished by the combinatorial expression of markers such as *S100A8, S100A9, LYZ, CD68, MSR1, CD163, CLEC4C, HLA-DRA, ITGAX, CD86, CD83*, and *MKI67*.

*Visualization and Analysis*:

UMAP: scanpy visualize the data using UMAP or t-SNE to explore the cellular landscape and identify distinct populations.

*Differential gene expression (DEG) analysis*:

For each gene, OLS regression models were fitted to normalized expression values, adjusting for sex, and age as covariates. For case-control comparisons, the model included disease status (case vs. control) as the primary variable of interest. For IOP analyses, only case samples were included, and IOP status (high vs. normal) was used as the main predictor. Analyses were performed both at the level of the entire B cell population and within each B cell subtype (subtypes with ≥50 cells for case-control and ≥30 cells for IOP analysis). Genes expressed in fewer than 10 cells per group were excluded from testing.

To improve computational efficiency, parallel processing was implemented using Python’s concurrent.futures and multiprocessing modules. P-values were adjusted for multiple testing using the Benjamini-Hochberg procedure to control the false discovery rate (FDR). Significant DEGs were defined as those with FDR < 0.05. All results were exported for downstream analysis and visualization.

Pathway analysis: To determine the changed levels of peripheral circulation molecules in patients with POAG, we identified pathways of DEGs (FDR<0.05) by comparing cell subtypes of POAG patients and healthy controls by gene enrichment analysis. We utilized the Python-based tool gseapy to perform DEG pathway enrichment analysis for KEGG and Reactome. We conducted separate analyses for upregulated DEG genes, downregulated DEG genes, and a combined analysis of both upregulated and downregulated DEG genes.

*PAGA Analysis of Pathway Activity in POAG PBMC Single-Cell Data*

PAGA (Partition-based Graph Abstraction) analysis was performed using scanpy (v1.9.0) based on unified cell subtypes, retaining only cell types with >1,000 cells. PAGA positions were computed using force-directed layout algorithms with UMAP cluster centroids as fallback.^1^ The resulting graph captured cellular hierarchy and transition probabilities.

Pathway Integration and Visualization

KEGG pathway enrichment analysis was performed for two comparisons: (1) Case versus Control, and (2) High IOP versus Normal IOP (case samples only). Pathway significance was determined using adjusted p-values (FDR < 0.05), with activity scores calculated as -log10(adjusted p-value). Top 5 most significant pathways per cell type were identified.

Network visualization integrated PAGA topology with pathway activity using custom matplotlib implementations. Node sizes were scaled logarithmically based on cell counts (40-150 units). Pathway markers were positioned using polar coordinates, with distinct shapes and colors for upregulated (circles, red/orange) and downregulated (squares, blue) pathways. Publication-ready figures used Arial font (8pt) with optimized color schemes for accessibility.

Statistical Analysis

Core pathways were identified by analyzing pathway overlap across comparison groups within each cell type, ranked by average significance score. Statistical significance was determined using adjusted p-values with FDR correction. All results were exported in multiple formats (PDF, PNG, CSV) with comprehensive logging for reproducibility. This PAGA analysis framework enables integration of single-cell transcriptomics with pathway enrichment data, facilitating identification of cell type-specific pathway alterations in complex disease contexts.

Random forest classifier predictions

We used scikit-learn random forest classifier^2^ for data prediction (hhttps://scikit-learn.org/stable/modules/generated/sklearn.ensemble.RandomForestClassifier.html). Feature extraction adopts the strategy of univariate statistics in automatic feature selection ^2^. In this study, a machine learning classification model based on random forest algorithm was constructed to analyze gene expression data. First, the original data set containing gene expression, gender, age and disease classification labels was imported from the excel file, and the classification variables were preprocessed by coding alone. The data set was divided into training set (49%), validation set (21%) and test set (30%) by stratified random sampling method to ensure that the distribution of sample categories in each subset was consistent with the original data set.

In the feature engineering stage, all features are used for model training without feature selection to ensure that the model can utilize all available information. The random forest classifier was used as the basic algorithm for model construction, and the grid search CV method was used to optimize the key hyper parameters, including the number of decision trees (n_estimators:80, 100) and the maximum tree depth (max_depth:10, 20, 40). In the optimization process, a 5-fold cross-validation was implemented to ensure the stability and generalization ability of the model.

The model evaluation adopts a multi-dimensional index system, including F1 score, ROC curve, AUC value, confusion matrix, and detailed classification report including accuracy and recall. By analyzing the importance of random forest characteristics, we can identify the key genes that have a significant impact on the classification results. To visually display the model performance, a standardized ROC curve is generated and a complete classification performance report is output. All prediction results are saved for further analysis.

In addition, to improve the reliability of the model and the stability of the results, the strategy of repeated experiments was designed in this study, and the number of experiments was controlled by setting the n_repeats parameter. Each experiment will be conducted independently to ensure the randomness and diversity of the results. By recording the results of each experiment, the performance fluctuation of the model under different experimental conditions can be analyzed, and the average and standard deviation of each performance index can be calculated to evaluate the overall performance and stability of the model.

Finally, the average ROC curve was drawn to comprehensively evaluate the performance of the model in all experiments. At the same time, the average feature importance of each feature in all experiments was calculated and ranked according to this average value to identify the key features that significantly affect the classification results. The AUC was calculated according to the true-positive rate and the false-positive rate, and the ROC curve was drawn accordingly. ^2^

This study ensures the reliability and reproducibility of the analysis results through strict cross-validation strategy and multiple evaluation indicators. This method not only provides accurate classification prediction but also provides an important basis for further understanding the role of key genes in signaling pathways.

*qPCR validation and pathway-specific machine learning analysis:*

To validate candidate factors identified from single-cell RNA sequencing (scRNA-seq) of PBMCs, we performed quantitative PCR (qPCR) on independent samples. Raw qPCR data were processed using the 2^-ΔΔCt method, and subsequently organized into a wide-format matrix with samples as rows and genes as columns. Missing values were imputed using the median of each feature. Case/control status, gender, and age were included as covariates, with gender encoded numerically.

For pathway-specific analysis, we focused on two immune signaling pathways: IFNG signaling (*IFNG*, *IFNGR1*, *IFNGR2, JAK1, JAK2, PTPN2, PTPN6, SOCS1, SOCS3, STAT1*) and TNF signaling (*CCL5, CREB5, ICAM1, IL1B, JUN, JUNB, MAPK13, SOCS3, TNF, TNFAIP3*). For each pathway, only genes detected in the qPCR dataset were included. Feature selection was performed using univariate ANOVA F-tests, and the top 10 features were retained for downstream modeling.

To assess the discriminative power of pathway-specific gene expression, we implemented an ensemble machine learning framework. For each pathway, the dataset was randomly split into training, validation, and test sets (70:21:9) in a stratified manner, and the analysis was repeated for 10 independent iterations to ensure robustness. Three ensemble classifiers—Random Forest, Gradient Boosting, and Extra Trees—were trained on the training set, with hyperparameters set to default values. Model performance was evaluated on the validation set using the F1 score, and the best-performing model was selected for final testing. All features were scaled using a robust scaler prior to model fitting.

Receiver operating characteristic (ROC) curves and area under the curve (AUC) values were computed for each iteration, and mean ROC curves were generated by interpolating true positive rates at 1,000 evenly spaced false positive rate points. Feature importance was extracted from the best-performing model in each iteration and averaged across repeats. For visualization, smoothed ROC curves and feature importance bar plots were generated for each pathway and iteration, as well as for the mean performance across all repeats. All analyses were performed in Python 3.8 using scikit-learn (v0.24), pandas (v1.2), numpy (v1.19), and matplotlib (v3.3). All code and scripts used for data processing, modeling, and visualization are available upon request.

Sample Genotyping

All the 110 POAG patients and 110 healthy controls were genotyped on the Axiom hNCG SNP array. CEL intensities were processed using the Analysis Power Tools

(APT) following the Axiom Best Practices Genotyping Analysis Workflow

(https://assets.thermofisher.com/TFSAssets/LSG/manuals/axiom_genotyping_solution_analysis_guide.pdf). 619,559 SNPs were retained for further analysis after evaluating for call rate, missingness, and heterozygosity with plink. After quality control, we retained 400,334 SNPs in the 205 samples with a minor allele frequency of greater than 0.05, HWE>e-6, mind 0.1. Finally, 295,132 SNPs with Rsq>0.3 were used for cis-eQTL analysis.

Genotype imputation

After quality control, we retained a total of 400,334 SNPs in the 205 samples; the SNPs had a minor allele frequency of over 0.05, HWE >×10^−6^, and mind < 0.1 (means that individuals with more than 10% missing genotype data will be excluded from the analysis) were used for imputation analysis on the CHN100K imputation server (http://bioinformatics.hit.edu.cn/imputation/toIndex) using Minimac4 method based on Chinese population (25,734 individuals). ^3^After data preocess, finally, 20,844,451 SNPs with MAF>0.01, Rsq > 0.3 were used for eQTL analysis.

eQTL Analysis

Building upon our discovery of extensive immune cell remodeling and pathway dysregulation in POAG PBMCs, we integrated genome-wide genotyping data with single-cell transcriptomic profiles to map cell type-specific expression quantitative trait loci (eQTLs) across five major PBMC populations. Genomic DNA was genotyped using [specify genotyping array/platform, e.g., Illumina Global Screening Array], and quality control was performed using PLINK v1.9, excluding SNPs and samples with high missingness (>5%), Hardy-Weinberg equilibrium P < 1×10^-6^, or minor allele frequency <1%. Genotype imputation was conducted using the Michigan Imputation Server with the [specify reference panel, e.g., 1000 Genomes Phase 3] as reference.

After QC and imputation, a total of 20,844,451 high-quality SNPs were retained for downstream analysis. Single-cell RNA-seq data were processed and aggregated by cell type and individual, generating pseudo-bulk expression matrices for each major PBMC subset. Expression values were normalized and log-transformed. eQTL mapping was performed using tensorQTL), ^4,5^ testing for both cis-eQTLs (variants within ±1 Mb of the transcription start site) and trans-eQTLs (variants located >1 Mb from the gene or on different chromosomes). Linear regression models were adjusted for age, sex, batch effects, and the top principal components of genetic ancestry to control for population stratification. Multiple testing correction was applied using the Benjamini-Hochberg procedure to control the false discovery rate (FDR < 0.05).

Performing Colocalization Analysis with SMR for eQTL and GWAS Data

SMR (Summary-based Mendelian Randomization)^6^ is a statistical method used to integrate eQTL (expression quantitative trait loci) and GWAS (genome-wide association study) data to identify genetic variants that may influence both gene expression and complex traits. Cis-eQTL analysis output summary data for POAG patients and GWAS summary data^7^ were used for SMR analysis.

Real-time quantitative polymerase chain reaction

Peripheral blood mononuclear cells (PBMCs) were isolated from fresh peripheral blood through density gradient centrifugation using Ficoll-Paque PLUS. Following collection in EDTA tubes, blood samples were diluted with PBS and layered onto Ficoll-Paque. The PBMC layer was harvested after centrifugation and washed to ensure cell purity. Total RNA was extracted using TRIzol reagent following the manufacturer's protocol, involving chloroform-based phase separation and isopropanol precipitation. The RNA pellet was washed with ethanol and reconstituted in RNase-free water. RNA quality was assessed using spectrophotometry and Bioanalyzer analysis, with samples meeting the quality threshold (RIN > 7) selected for further analysis. First-strand cDNA synthesis was performed using a reverse transcription kit with random primers under optimized thermal conditions. Gene expression was quantified using SYBR Green-based real-time PCR on an Applied Biosystems 7500 Fast Real-time PCR System (Thermo Fisher Scientific, USA) with PerfectStart Green qPCR SuperMix (TransGen Biotech, China). The following primers were used:

- β-Actin: Forward 5'-GAAGATCAAGATCATTGCTCCT-3'

Reverse 5'-TACTCCTGCTTGCTGATCCA-3'

- TNFα: Forward 5'-AGGCGGTGCTTGTTCCTC-3'

Reverse 5'-GGCTTGTCACTCGGGGTT-3'

All samples were analyzed in technical duplicates. Relative expression levels were calculated using the 2^(-ΔΔCt) method, with β-Actin serving as the internal reference gene. Statistical analysis was performed using the Student's t-test between the POAG group (n=45) and the control group (n=43). Data are presented as mean ± SEM, with statistical significance set at p<0.0001.

**Animals and Experimental Model of Retinal Injury**

All animal procedures were approved by the Institutional Animal Care and Use Committee of Sichuan Academy of Medical Sciences and Sichuan Provincial People's Hospital and conducted in accordance with the ARVO Statement for the Use of Animals in Ophthalmic and Vision Research. Wild-type (WT) C57BL/6J mice, *Ifng* knockout (*Ifng*^-/-^) mice (S-KO-16487_6N_15978_Ifng from Cyagen), and Tnf heterozygous (*Tnf*^+/-^) mice (S-KO-17975_6J_21926_Tnf from Cyagen), all on a C57BL/6J background and aged 8-10 weeks, were used in this study.

To induce experimental retinal injury mimicking aspects of glaucoma, mice first received an intraperitoneal (i.p.) injection of lipopolysaccharide (LPS; 0.2 mg/kg; Sigma-Aldrich) dissolved in sterile saline. Two hours following LPS administration, mice were anesthetized via i.p. injection of 2.5% Tribromoethanol (Avertin; working concentration administered at 0.1 ml/10 g body weight; T48402-25G, Sigma-Aldrich). Pupils were dilated using 1% tropicamide ophthalmic solution. Under a dissection microscope, 1.5 µL of 5 mM N-methyl-D-aspartate (NMDA; Sigma-Aldrich) dissolved in sterile PBS was carefully injected into the vitreous cavity of each eye using a 5 µl Microliter Syringe (PN: 7634-01; Hamilton, USA). Immediately after injection, levofloxacin ophthalmic ointment was applied to the ocular surface of both eyes to prevent potential bacterial infection. Animals were housed under standard conditions and euthanized for tissue collection and analysis 48 hours post-NMDA injection.

**Retinal Wholemount Preparation and Immunofluorescence**

Mice were euthanized by cervical dislocation. Eyes were immediately enucleated and fixed in 4% paraformaldehyde (PFA) in phosphate-buffered saline (PBS, pH 7.4) on ice for 30 minutes. Subsequently, eyes were transferred to fresh cold PBS and incubated on ice for an additional 30 minutes. The retinas were then carefully dissected from the eyecup, and four radial incisions were made from the edge towards the optic nerve head to create a flattened 'petal' configuration. The dissected retinas were then immersed in ice-cold 100% methanol and stored at -20°C overnight for post-fixation and permeabilization.

The following day, retinas were rehydrated and washed three times with PBS for 5 minutes each at room temperature. Non-specific binding sites were blocked by incubating the retinas for 2 hours at room temperature in blocking buffer consisting of 10% Fetal Bovine Serum (FBS), 0.25% Triton X-100, and 0.03% sodium azide in PBS. Retinas were then incubated overnight at 4°C with primary antibody against the retinal ganglion cell marker Brn3a (Rabbit anti-Brn3a, 1:300 dilution; ab245230, Abcam). After primary antibody incubation, retinas were washed three times with PBS (5 min each) and subsequently incubated for 2 hours at room temperature with Alexa Fluor™ 488 conjugated goat anti-rabbit IgG secondary antibody (1:1000 dilution; A-11008, Invitrogen). Following three final washes with PBS (5 min each), the retinas were carefully mounted flat onto glass slides using an aqueous anti-fade mounting medium (e.g., VectaShield or ProLong Gold). Images were acquired using a Zeiss LSM900 confocal laser scanning microscope (Carl Zeiss, Germany).

*Retinal Cryosection Preparation and Immunofluorescence*

Mice were euthanized by cervical dislocation, and eyes were enucleated and fixed in 4% PFA in PBS on ice for 10 minutes. A small puncture was carefully made near the limbus with a fine needle to facilitate fixative penetration, and eyes were then further fixed in 4% PFA on ice for 2 hours. After fixation, eyes were washed three times with PBS (5 min each) and cryoprotected by immersion in 30% sucrose in PBS at 4°C for at least 4 hours or until the tissue sank. Eyes were then embedded in Optimal Cutting Temperature (OCT) compound (Tissue-Tek) in cryomolds and rapidly frozen.

Frozen blocks were sectioned at a thickness of 12 µm using a cryostat. Sections containing or immediately adjacent to the optic nerve head were collected onto adhesive microscope slides (G6012-1, Servicebio). Slides were air-dried in a 37°C oven for 30 minutes and then washed three times with PBS (5 min each). Sections were permeabilized and blocked for 1 hour at room temperature in blocking buffer (10% FBS, 0.25% Triton X-100, 0.03% sodium azide in PBS). Sections were then incubated overnight at 4°C with one or a combination of the following primary antibodies diluted in blocking buffer:

Brn3a (Rabbit anti-Brn3a, 1:300; ab245230, Abcam)

GFAP (Rabbit anti-GFAP, 1:300; #80788, Cell Signaling Technology)

IBA1/AIF1 (Rabbit anti-IBA1, 1:200; A19776, Abclonal)

PSD95 (Rabbit anti-PSD95, 1:200; A0131, Abclonal)

Synaptophysin (Mouse anti-Synaptophysin, 1:300; 17785-1-AP, Proteintech)

CaMKII (Mouse anti-CaMKII, 1:300; PA5-19128, Invitrogen)

βIII-tubulin (Rabbit anti-βIII-tubulin, 1:300; A17913, Abclonal)

OPA1 (Rabbit anti-OPA1, 1:200; A9833, Abclonal)

DRP1 (Rabbit anti-DRP1, 1:100; A21968, Abclonal)

FIS1 (Rabbit anti-FIS1, 1:100; 10956-1-AP, Proteintech)

Following primary antibody incubation, slides were washed three times with PBS (5 min each). Sections were then incubated for 1 hour at room temperature with appropriate Alexa Fluor™ 488(Goat anti-Rabbit IgG (H+L) Cross-Adsorbed Secondary Antibody; A-11008, 1:1000 dilution; Invitrogen) or Alexa Fluor™ 594 conjugated secondary antibodies (Goat anti-Mouse IgG (H+L) Cross-Adsorbed Secondary Antibody; A-11005, 1:1000 dilution; Invitrogen) and the nuclear counterstain DAPI (4′,6-diamidino-2-phenylindole, 1:1000 dilution; Solarbio, China) diluted in PBS. After incubation, slides were washed three times with PBS (5 min each) and coverslipped using aqueous anti-fade mounting medium. Images were acquired using a Zeiss LSM900 confocal microscope and processed using Zen 3.6 software (Carl Zeiss). Fluorescence intensity and cell counts were quantified using software ImageJ/Fiji.

*Flash Electroretinography (ERG) and Visual Evoked Potentials (VEP)*

Scotopic flash ERG and flash VEP recordings were performed using the Celeris Electrodiagnostic System (Diagnosys Ltd, Lowell, MA, UK). Mice were dark-adapted overnight (at least 12 hours) before the procedure. All subsequent steps until recording completion were performed under dim redlight illumination (< 1 lux). Mice were anesthetized using an i.p. injection of 2.5% Tribromoethanol (0.1 ml/10 g). Pupils were dilated with 1% tropicamide. Body temperature was maintained using a heating pad.

For ERG recordings, gold-wire loop electrodes were placed on the cornea of both eyes after applying a drop of hypromellose solution for lubrication and electrical contact. Reference electrodes were inserted subcutaneously near the temporal canthus, and a ground electrode was inserted subcutaneously in the tail. Scotopic flash ERG responses were recorded simultaneously from both eyes in response to a standard flash intensity of 1.0 cd·s/m².

For VEP recordings, following placement of the corneal electrodes (active), three stainless steel needle electrodes were inserted subcutaneously: one over the visual cortex (active, approximately 3 mm lateral to lambda), one over the contralateral prefrontal cortex (reference), and one in the tail (ground). VEP responses were averaged from multiple flash presentations (50 flashes).

After recordings, electrodes were removed, and levofloxacin ophthalmic ointment was applied to both eyes. Amplitudes (ERG a-wave(N-A), b-wave(N-B); VEP N1-P1) and implicit times were measured and analyzed.

*Light-Dark Box Test*

Anxiety-like behavior and photophobia were assessed using the light-dark box test (LDBT) apparatus (Model XR-XB110, Shanghai Xinruan Information Technology Co., Ltd.). The apparatus consists of two compartments, one brightly lit (~2000 lux) and one dark (< 5 lux), connected by a small opening. Mice were first habituated to the testing room environment for at least 30 minutes prior to testing. For each trial, a mouse was initially placed into the center of the dark compartment, facing away from the opening. The partition between the compartments was then removed, and the mouse was allowed to freely explore the apparatus for 5 minutes. Locomotor activity, transitions between compartments, and time spent in each compartment were automatically recorded and tracked using an overhead video camera and associated analysis software. The apparatus was thoroughly cleaned with 75% ethanol between each trial to eliminate olfactory cues. Data collected included total distance traveled, time spent in the light compartment (%), number of transitions, and latency to first enter the light compartment. Trajectory heatmaps were also generated.

*Flow Cytometry Analysis of Peripheral Blood Immune Cells*

Peripheral blood was collected via cardiac puncture immediately following euthanasia into tubes containing heparin sodium anticoagulant. Staining and analysis were performed using whole blood lysis protocols. 10× RBC Lysis Buffer (E-CK-A105, Elabscience) and 10× Permeabilization Buffer (IC001-100, MultiSciences) were diluted to 1× working concentration with distilled water as needed. 1× Flow Cytometry Staining Buffer (S1001, MultiSciences) was used for washes and final resuspension unless otherwise noted.

Panel 1: Intracellular IFN-γ in T cells:

1. 125 µl of heparinized blood was mixed with 125 µl of serum-free RPMI medium in a flow tube.

2. For stimulation, 1 µl of PMA/Ionomycin Mixture (250×, HY-18739, MCE) and 1 µl of BFA/Monensin Mixture (250×, HY-N0150, MCE) were added. An unstimulated control tube received only blood and medium.

3. Tubes were mixed gently and incubated at 37°C in a 5% CO2 incubator for 4-6 hours, with gentle mixing every 1-2 hours.

4. 100 µl of stimulated or control blood was transferred to new tubes.

5. Surface staining: Add 5 µl ER780 Anti-Mouse CD3 Antibody [17A2] (E-AB-F1013S, Elabscience) and 5 µl PerCP/Cyanine5.5 Anti-Mouse CD8a Antibody [53-6.7] (E-AB-F1104J, Elabscience). Mix and incubate at room temperature (RT) in the dark for 15 minutes.

6. RBC Lysis: Add 2 ml of 1× RBC Lysis Buffer, mix, and incubate at 4°C in the dark for 5-10 minutes.

7. Stop lysis by adding 3 ml PBS. Centrifuge at 300-400 × g for 5 minutes at 4°C. Discard supernatant.

8. Fixation: Add 100 µl FIX & PERM™ Medium A (Invitrogen), mix, and incubate at RT in the dark for 15 minutes.

9. Wash: Add 2 ml pre-cooled 1× Flow Cytometry Staining Buffer, centrifuge at 300 × g for 5 minutes. Discard supernatant.

10. Permeabilization & Intracellular Staining: Add 100 µl FIX & PERM™ Medium B (Invitrogen) and 5 µl Anti-Mouse IFN gamma(t) FITC (11-7311-82, eBioscience). Mix and incubate at RT in the dark for 15 minutes.

11. Wash: Add 2 ml 1× Flow Cytometry Staining Buffer, centrifuge at 300 × g for 5 minutes. Discard supernatant.

12. Resuspend pellet in 500 µl 1× Flow Cytometry Staining Buffer for immediate acquisition or in 500 µl 1-4% PFA for storage at 2-8°C (analyze within 24 hours).

13. Acquisition: Use appropriate laser/filter settings (CD3: Red laser(528), APC-A750 filter; CD8a: Blue laser(488), PerCP-Cy5.5 filter; IFN-γ: Blue laser(488), FITC filter).

Panel 2: FOXP3 in Regulatory T cells (Tregs):

1. Prepare 1× Fixation/Permeabilization working solution by mixing Fixation/Permeabilization Concentrate (4×) and Fixation/Permeabilization Diluent (IC001-100, MultiSciences; Foxp3 / Transcription Factor Staining Buffer Set) at a 1:3 ratios. Prepare 1× Permeabilization Buffer from 10× stock.

2. Surface staining: To 100 µl heparinized blood, add 5 µl FITC Anti-Mouse CD4 Antibody [GK1.5] (E-AB-F1097C, Elabscience) and 0.625 µl APC Anti-Mouse CD25 Antibody [PC-61.5.3] (E-AB-F1102E, Elabscience). Mix and incubate at 4°C in the dark for 30-60 minutes.

3. RBC Lysis: Add 2 ml of 1× RBC Lysis Buffer, mix, and incubate at 4°C in the dark for 5-10 minutes.

4. Stop lysis by adding up to 10 ml PBS (or fill tube). Centrifuge at 300-400 × g for 5 minutes at 4°C. Discard supernatant.

5. Fixation/Permeabilization: Add 1 ml of freshly prepared 1× Fixation/Permeabilization working solution. Mix well and incubate at RT in the dark for 30-60 minutes.

6. Wash: Add 2 ml 1× Permeabilization Buffer. Centrifuge at 300-400 × g for 5 minutes at 4°C. Discard supernatant.

7. Repeat wash step (Step 6).

8. Resuspend pellet in 100 µl 1× Permeabilization Buffer.

9. Block: Add 2 µl Normal Rat Serum (AB_2337141, Jackson ImmunoResearch) and incubate at RT in the dark for 15 minutes.

10. Intracellular Staining: Without washing, add 5 µl Anti-Mouse FOXP3 (12-5773-82, eBioscience; Clone FJK-16s, conjugated to PE). Mix and incubate at RT in the dark for at least 30 minutes.

11. Wash: Add 2 ml 1× Permeabilization Buffer. Centrifuge at 300-400 × g for 5 minutes at RT. Discard supernatant.

12. Repeat wash step (Step 11).

13. Resuspend pellet in 500 µl 1× Flow Cytometry Staining Buffer for acquisition.

14. Acquisition: Use appropriate laser/filter settings (CD4: Blue laser (488), FITC filter; CD25: Red laser (538), APC filter; FOXP3: Blue laser (488), PE filter).

Panel 3: General Lymphocyte and NK cell Markers:

1. Surface staining: To 100 µl heparinized blood, add 5 µl each of: Elab Fluor® Red 780 Anti-Mouse CD3 Antibody (E-AB-F1013S, Elabscience), FITC Anti-Mouse CD4 Antibody (E-AB-F1097C, Elabscience), PerCP/Cyanine5.5 Anti-Mouse CD8a Antibody (E-AB-F1104J PerCP, Elabscience), PE/Cyanine7 Anti-Mouse CD19 Antibody[1D3] (E-AB-F0986H, Elabscience), and APC Anti-Mouse CD161/NK1.1 Antibody (E-AB-F0987E, Elabscience). Mix well and incubate at RT in the dark for 15-30 minutes (or 30-60 minutes on ice).

2. RBC Lysis: Add 2 ml of 1× RBC Lysis Buffer, mix, and incubate on ice in the dark for 10 minutes. Centrifuge at 300 × g for 5 minutes. Discard supernatant.

3. Wash: Add 2 ml PBS, mix, centrifuge at 300 × g for 5 minutes. Discard supernatant.

4. Resuspend pellet in 500 µl 1× Flow Cytometry Staining Buffer for acquisition.

Flow cytometry data were acquired on a [CytoFLEX, Beckman coulter] and analyzed using FlowJo software (Version X, BD Life Sciences). Gating strategies involved initial gating on lymphocytes based on FSC-A vs SSC-A, followed by doublet exclusion (FSC-H vs FSC-W and SSC-H vs SSC-W), and then identification of specific populations based on marker expression (e.g., CD3⁺ for T cells, CD19⁺ for B cells, CD3⁻NK1.1⁺ for NK cells, CD3⁺CD4⁺ or CD3⁺CD8⁺ for T cell subsets, CD4⁺CD25⁺FOXP3⁺ for Tregs).

*Western blot analyses*

Blood was collected from the anesthetized mice via the abdominal aorta and retina. Peripheral blood mononuclear cells (PBMC) were isolated from whole blood using Ficoll-Paque PLUS lymphocyte separation medium (Cat. No. 17144002, Cytiva). RIPA lysis buffer supplemented with protease and phosphatase inhibitors was added to the PBMC and retinal tissues, followed by sonication on ice. Protein concentration was determined using the Coomassie Brilliant Blue method. Protein samples were loaded onto either a 12.5% or 15% SDS-PAGE gel and separated by electrophoresis, then transferred onto a 0.45 μm nitrocellulose (NC) membrane (Cat. No. WJ004, Epizyme Biotech). The membrane was blocked with 8% skimmed milk powder at room temperature for 1.5 hours and subsequently incubated with primary antibody overnight at 4 °C. After washing with TBST, membranes were incubated with HRP-conjugated secondary antibody at room temperature for 1 hour. After additional TBST washing, membranes were treated with ECL chemiluminescent substrate solution (Cat. No. WBULS0100, Millipore) and exposed for detection.

Primary antibodies were diluted as follows: FOXP3 1:2000 (cat.no. A5706, Abclonal); CD8a 1:2000 (cat.no. 29896-1-AP, Proteintech); CD4 1:2000 (cat.no. A26036PM, Abclonal); CaMKII 1:1000 (cat.no. A25000, Abclonal); Phospho-CaMKII (Thr286) 1:2000 (cat.no. #A25000, Cell Signaling Technology); OPA1 1:1000 (cat.no. A9833, Abclonal); DRP1 1:1000 (cat.no. A21968, Abclonal); FIS1 1:2000 (cat.no. 10956-1-AP, Proteintech); MT-ATP6 1:1000 (cat.no. A17960, Abclonal); MTCO2 1:2000 (cat.no. 55070-1-AP, Proteintech); TOM20 1:2000 (cat.no. 10956-1-AP, Proteintech); β-actin 1:10000 (cat.no. AC026, Abclonal). Blots were quantified by ImageJ software (https://imagej.nih.gov/ij/).

*Total RNA isolation and cDNA synthesis*

The whole blood and retina were used to extract the total RNA. The samples were homogenized, and then the total RNA was isolated using Trizol reagent (cat.no. RK30129, Abclonal). Reverse transcription into cDNA was performed using ABScript Neo RT Master Mix for qPCR with gDNA Remover Kit (cat.no. RK20433, Abclonal).

Real-time quantitative polymerase chain reaction

The RT-qPCR was performed on the Applied Biosystems PCR System (Thermo Fisher Scientific, USA) using BrightCycle Universal SYBR Green qPCR Mix with UDG (cat.no. RK21219, Abclonal). The PCR amplification was repeated thrice for each sample. Relative gene expression was calculated using the 2-ΔΔCt method, normalizing to β-actin (ACTB) as the housekeeping gene.

*Statistical Analysis*

Data are presented as mean ± standard deviation (SD) or standard error of the mean (SEM) as indicated in the figure legends. Statistical comparisons between two groups were performed using unpaired two-tailed Student's t-tests. Comparisons involving more than two groups were performed using one-way analysis of variance (ANOVA) followed by an appropriate post-hoc test (Tukey's or Dunnett's multiple comparisons test). Statistical significance was set at *P* < 0.05. All statistical analyses were performed using GraphPad Prism version 6.


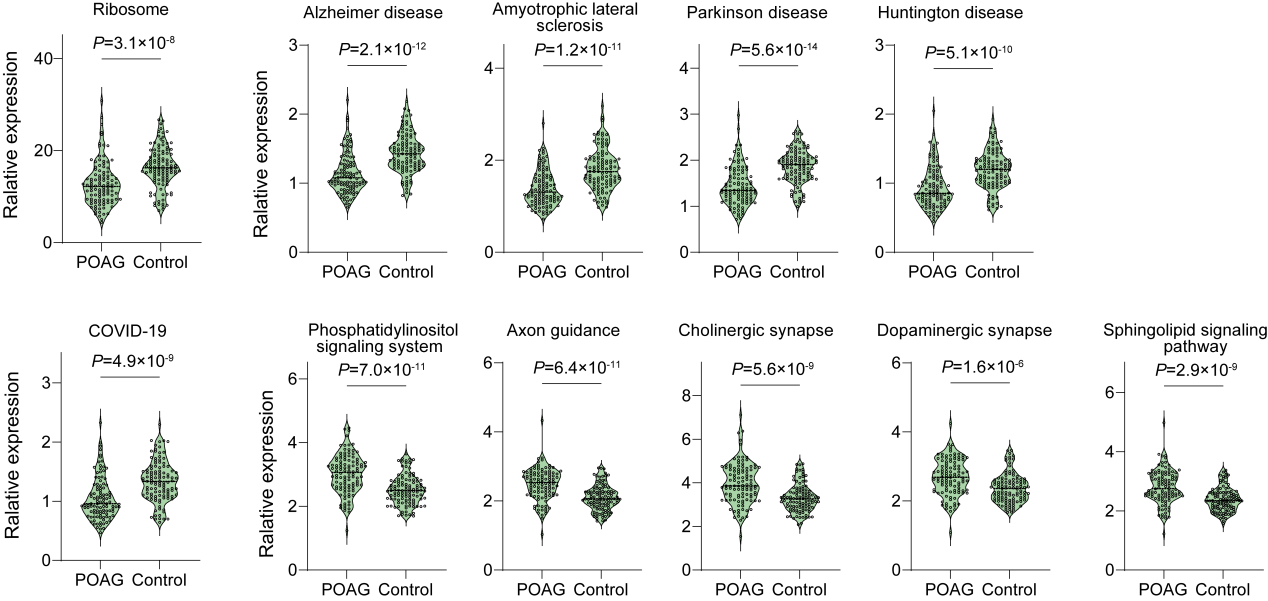


**Supplementary Fig. 1.Pathway enrichment analysis identifies distinct transcriptional signatures in POAG.** Violin plots showing relative total expression of selected pathway genes and disease-related gene sets in PBMCs from POAG patients compared to controls. Neurodegenerative disease pathways (Alzheimer disease, Parkinson disease, Huntington disease, Amyotrophic lateral sclerosis) and COVID-19 show significantly downregulated expression in POAG patients, while pathways related to phosphatidylinositol signaling, axon guidance, and synaptic function show upregulated expression. These findings suggest that POAG patients exhibit altered expression of pathways associated with neurodegeneration and synaptic function. All comparisons show highly significant differences (*P* < 1.6×10⁻⁶). Data are presented as mean ± SEM.

**
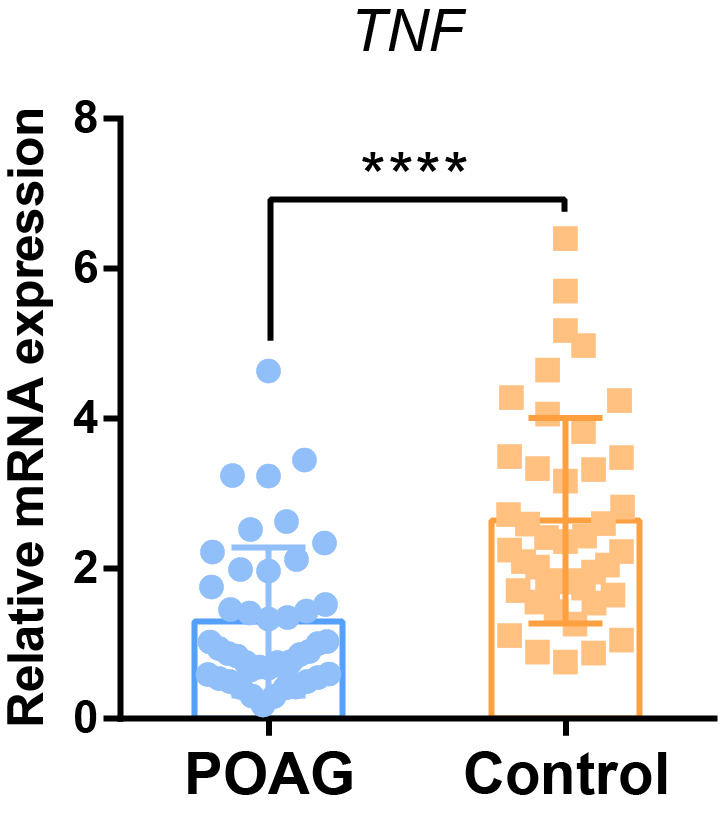
**

**Supplementary Fig. 2. qPCR validation of *TNF* expressin in another cohort of 45 POAG and 43 controls.**


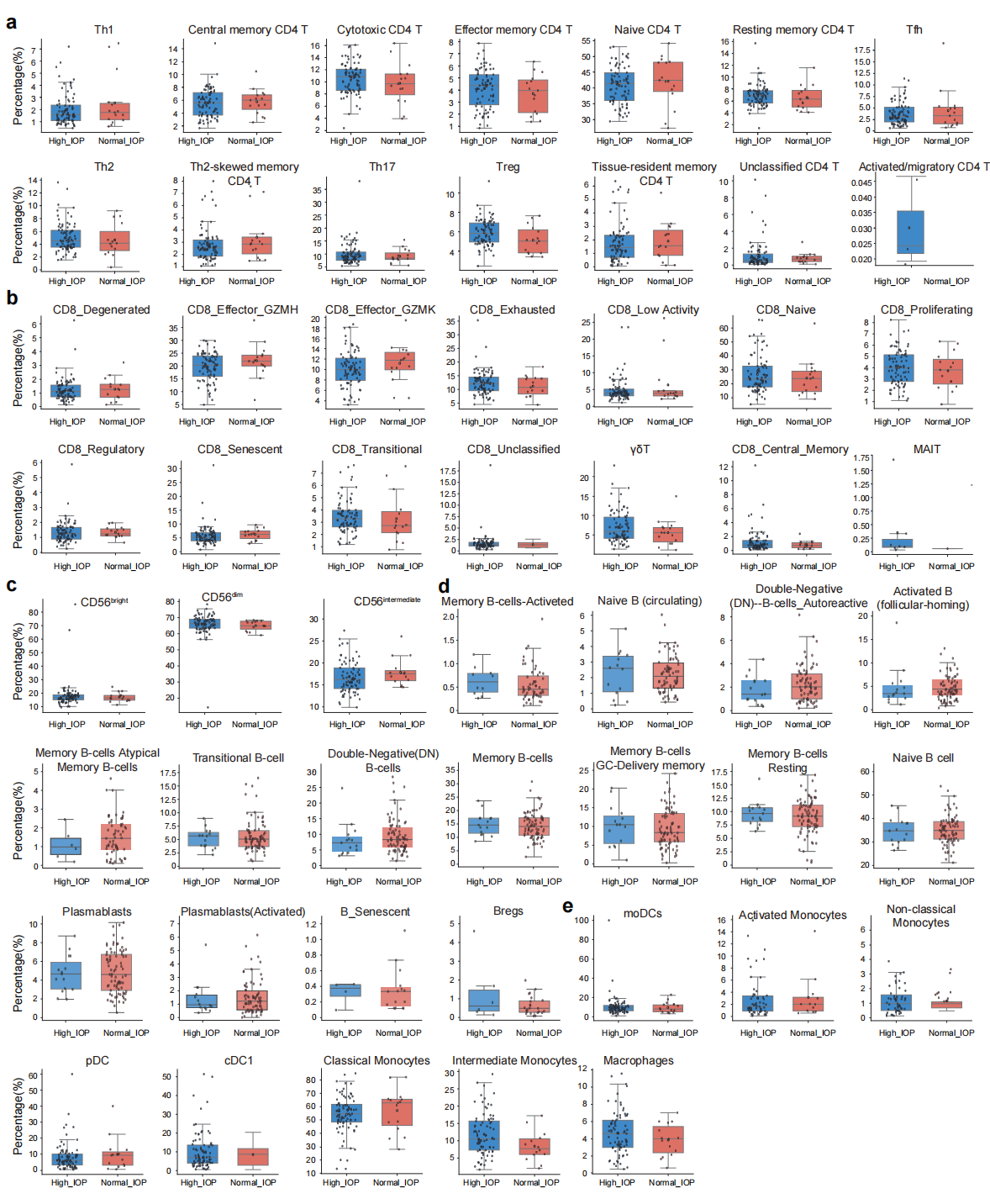


**Supplementary Fig. 3. Immune cell subtype composition in PBMCs of POAG patients stratified by IOP.** Analysis of PBMC cell subtypes and subtypes revealed no statistically significant compositional differences between the high-IOP and normal-IOP groups. **a** CD4^+^ T cell subtypes, **b** CD8^+^ T cell subtypes, **c** Natural Killer (NK) cell subtypes, **d** B cell subtypes, and **e** Monocyte and Dendritic cell (DC) subtypes.


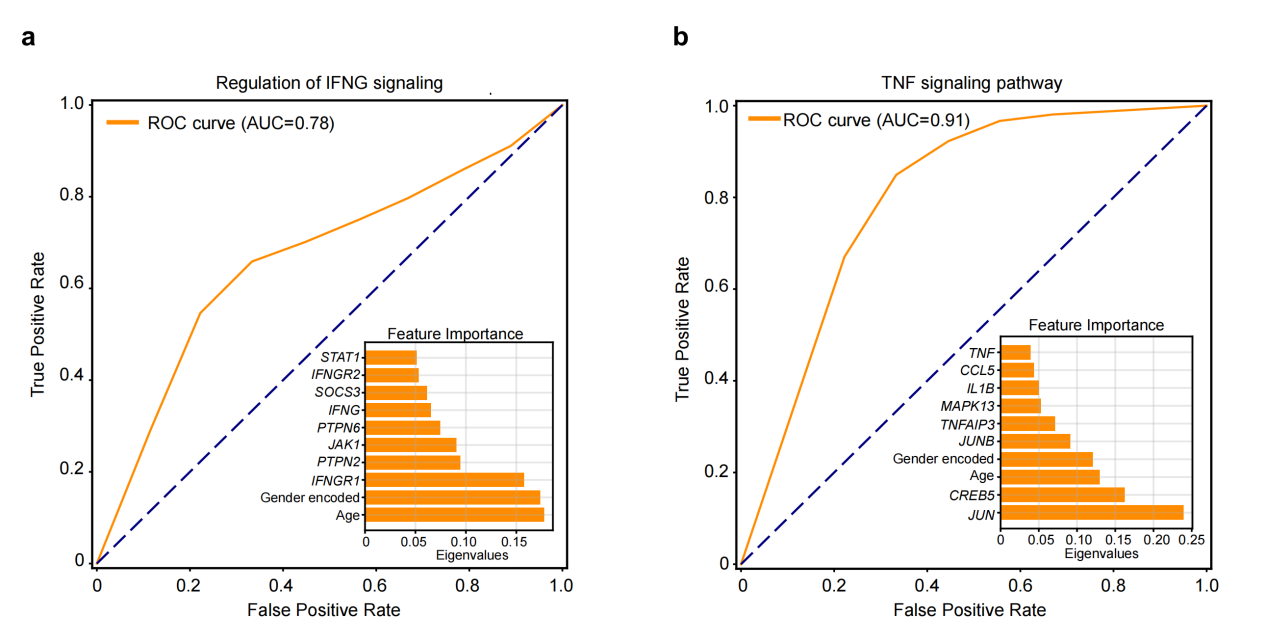


**Supplementary Fig. 4. Predictive performance of pathway-specific gene expression signatures for POAG case-control status based on qPCR validation.** We conducted independent validation using quantitative polymerase chain reaction (qPCR) on a separate cohort of 59 samples (31 cases, 28 controls). **a** Receiver operating characteristic (ROC) curve for the IFNG signaling pathway, with an area under the curve (AUC) of 0.78, demonstrating the ability of IFNG pathway gene expression to discriminate between POAG cases and controls. The inset bar plot shows the relative importance of each feature (gene, gender, and age) in the optimized ensemble model, with *STAT1, IFNGR2,* and *SOCS3* contributing most to the classification. **b** ROC curve for the TNF signaling pathway, with an AUC of 0.91, indicating high predictive accuracy for case-control status. The corresponding feature importance plot highlights *JUN, CREB5*, and age as the top contributors in the TNF pathway model. Both models were trained and evaluated using repeated cross-validation and ensemble machine learning approaches on qPCR-validated gene expression data from PBMCs. See Methods for details.

| **Cell_Type** | **eQTL_Type** | **Condition** | **Total_SNPs** | **LD_Count** | **Total_Genes** | **Significant_SNPs** | **Significant_Genes** | **Significant_LD_Groups** | **Significant_Large_LD_Groups** |
| --- | --- | --- | --- | --- | --- | --- | --- | --- | --- |
| CD4 | cis | Control | 19243 | 5220 | 19243 | 19149 | 19149 | 419 | 262 |
| CD4 | cis | POAG | 19259 | 5335 | 19259 | 19183 | 19183 | 425 | 251 |
| CD4 | trans | Control | 387937 | 4666 | 7582 | 387937 | 7582 | 471 | 424 |
| CD4 | trans | POAG | 132186 | 4321 | 5143 | 132186 | 5143 | 538 | 459 |
| CD8 | cis | Control | 19245 | 5254 | 19245 | 19136 | 19136 | 403 | 255 |
| CD8 | cis | POAG | 19232 | 5391 | 19232 | 19120 | 19120 | 390 | 243 |
| CD8 | trans | Control | 547157 | 4814 | 8239 | 547157 | 8239 | 426 | 391 |
| CD8 | trans | POAG | 157119 | 4652 | 5524 | 157119 | 5524 | 476 | 407 |
| NK | cis | Control | 19214 | 5186 | 19214 | 19106 | 19106 | 436 | 260 |
| NK | cis | POAG | 19228 | 5390 | 19228 | 19116 | 19116 | 378 | 245 |
| NK | trans | Control | 572945 | 4615 | 8623 | 572945 | 8623 | 480 | 428 |
| NK | trans | POAG | 198811 | 4765 | 6116 | 198811 | 6116 | 423 | 372 |
| B_cell | cis | Control | 19226 | 5275 | 19226 | 19118 | 19118 | 426 | 273 |
| B_cell | cis | POAG | 19224 | 5302 | 19224 | 19120 | 19120 | 418 | 257 |
| B_cell | trans | Control | 384446 | 4770 | 7662 | 384446 | 7662 | 420 | 383 |
| B_cell | trans | POAG | 214511 | 4520 | 6282 | 214511 | 6282 | 473 | 410 |
| myeloid | cis | Control | 19224 | 5259 | 19224 | 19112 | 19112 | 419 | 255 |
| myeloid | cis | POAG | 19218 | 5355 | 19218 | 19107 | 19107 | 402 | 245 |
| myeloid | trans | Control | 378341 | 4998 | 7564 | 378341 | 7564 | 385 | 351 |
| myeloid | trans | POAG | 258672 | 4860 | 7742 | 258672 | 7742 | 414 | 374 |

**Supplementary Fig. 5.** Summary of eQTL discovery across major PBMC cell types in POAG and control groups. The table summarizes the number of total and significant eQTLs identified in cis and trans analyses for each major immune cell type (CD4⁺ T cells, CD8⁺ T cells, NK cells, B cells, and myeloid cells) under POAG and control conditions. For each cell type and condition, the total number of tested SNPs, LD (linkage disequilibrium) groups, and genes are shown, along with the number of significant SNPs, significant genes, significant LD groups, and significant large LD groups (defined by group size). Notably, the number of significant eQTLs and LD groups varies between POAG and control samples, and between cis and trans analyses, reflecting both disease-specific and cell type-specific regulatory landscapes. See Methods for statistical thresholds and definitions.


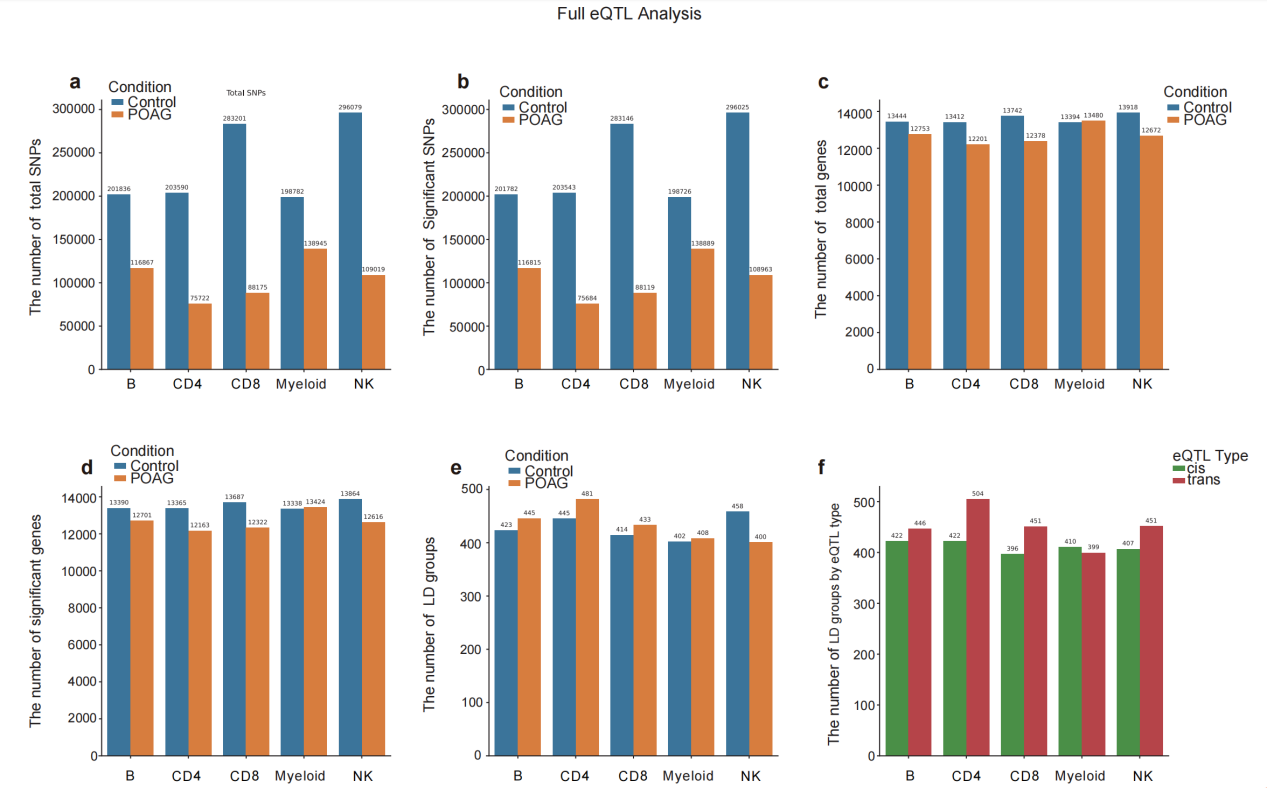


**Supplementary Fig. 6. Comparative eQTL landscape in major immune cell types between POAG cases and controls. a** Bar plot showing the total number of SNPs tested for eQTLs in B cells, CD4^+^ T cells, CD8^+^ T cells, myeloid cells, and NK cells, for POAG cases (orange) and controls (blue). **b** Bar plot showing the number of significant SNPs (FDR < 0.05) identified as eQTLs in each cell type for cases and controls. **c** Bar plot showing the total number of genes tested for eQTLs in each cell type for cases and controls. **d** Bar plot showing the number of significant genes (FDR < 0.05) identified as eQTL targets in each cell type for cases and controls. **e** Bar plot showing the number of LD groups identified in each cell type for cases and controls. **f** Bar plot showing the number of LD groups stratified by eQTL type (cis: green, trans: red) in each cell type (combined for cases and controls). For panels **a**-**e**, orange bars represent POAG cases and blue bars represent controls. For panel **f**, green bars represent cis-eQTLs and red bars represent trans-eQTLs.

**
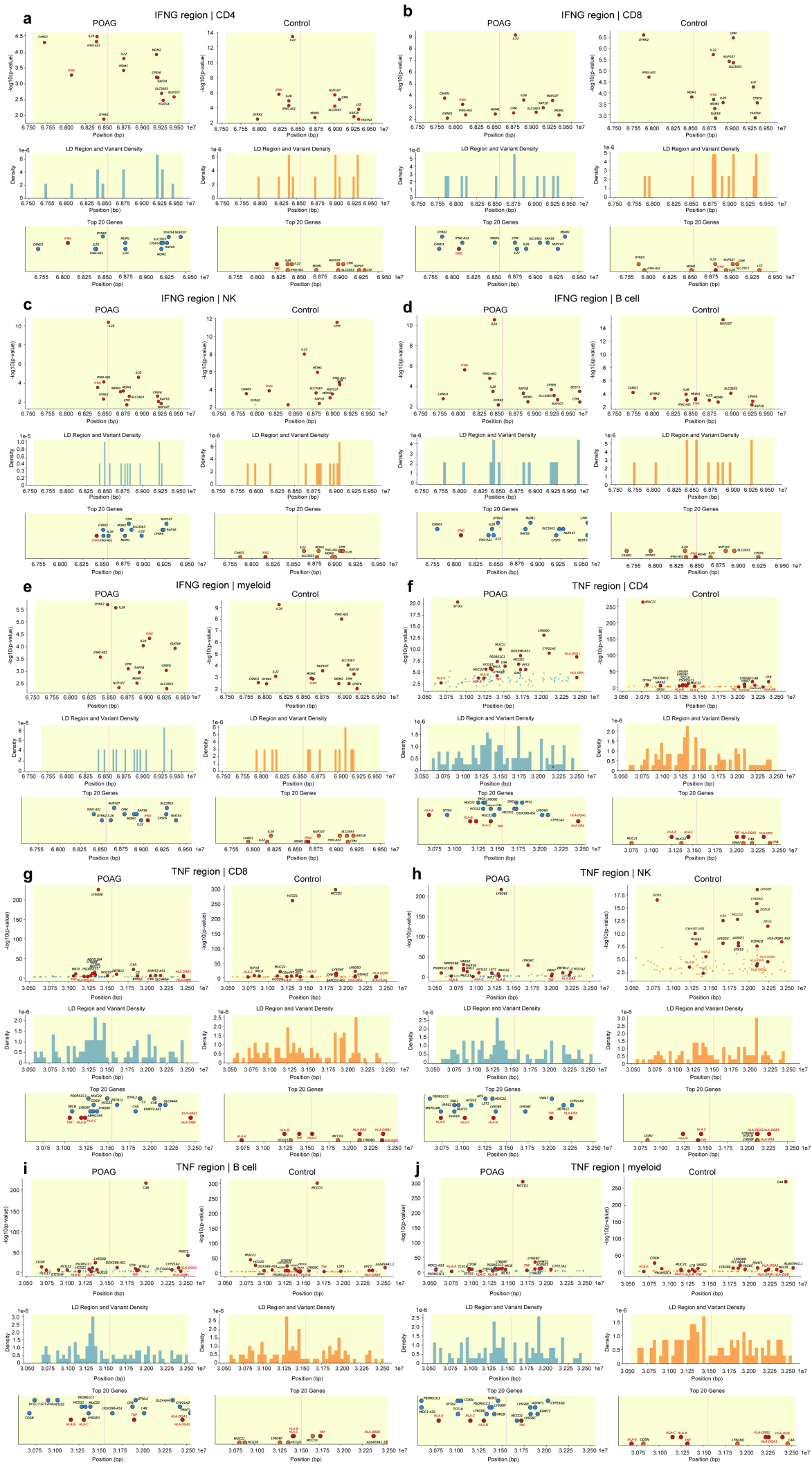
**

**Supplementary Fig. 7. Fine-mapping of eQTLs in the IFNG and TNF genomic regions across major PBMC subsets in POAG and controls.** Regional eQTL association plots for the IFNG (**a**–**e**) and TNF (**f**–**j**) loci are shown for CD4⁺ T cells, CD8⁺ T cells, NK cells, B cells, and myeloid cells, comparing POAG cases (left panels) and controls (right panels). For each cell type and locus, the top panels display the –log₁₀(P) values of eQTL associations across the genomic region, highlighting the distribution and intensity of significant regulatory variants. Middle panels depict the density of variants and linkage disequilibrium (LD) groups, while bottom panels show the top 20 eQTL target genes ranked by association strength and genomic position. Striking differences in eQTL architecture were observed between POAG and control samples at both loci. In the IFNG region (**a**–**e**), POAG samples exhibited a broader and more pronounced spectrum of significant eQTL associations across all immune cell types, with several variants exerting stronger regulatory effects on IFNG and neighboring immune-related genes. Control samples, in contrast, displayed a more restricted and less intense eQTL landscape. Similarly, at the TNF locus (**f**–**j**), POAG samples demonstrated a substantial increase in both the number and magnitude of significant eQTLs affecting TNF and adjacent cytokine genes, including LTA and LTB, across all cell types. The divergence in regulatory profiles was particularly marked in CD4⁺ T cells and myeloid cells, where POAG-specific eQTL hotspots were evident. These findings suggest enhanced and cell type-specific genetic regulation of key immune pathways in POAG.

**
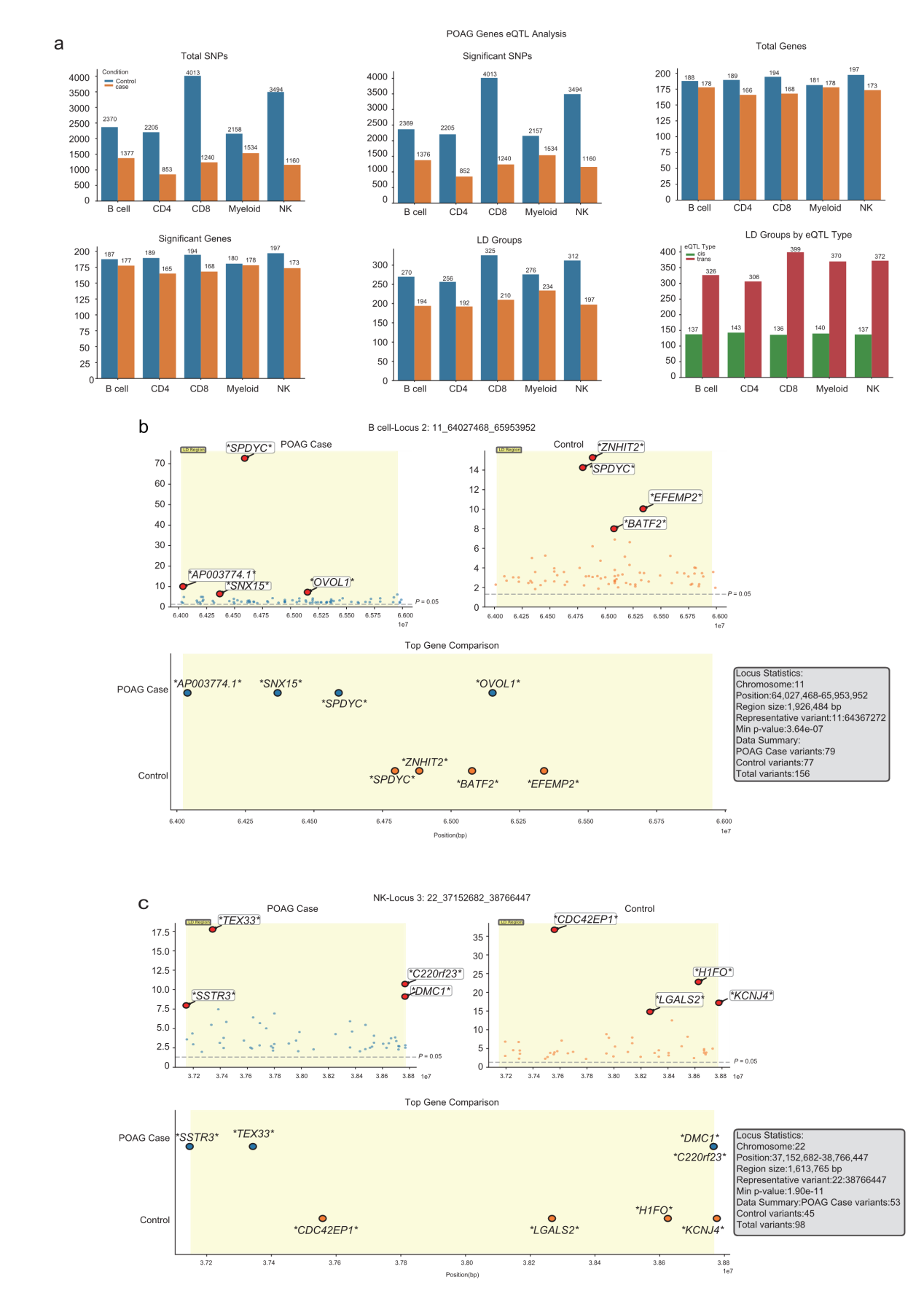
**

**Supplementary Fig. 8. eQTL mapping at single-cell resolution for reported POAG GWAS Gene loci.** **a** Quantitative summary of eQTL mapping results across major PBMC cell types, showing the total and significant numbers of SNPs, genes, and LD groups identified in POAG cases and controls. Both the number of significant SNPs and regulated genes varied substantially by cell type, with myeloid cells and CD4⁺ T cells exhibiting the highest counts of significant eQTLs and LD groups. **b** Locus-level analysis of a representative B cell eQTL region on chromosome 11 reveals marked regulatory divergence between POAG and control samples. In POAG cases, strong eQTL signals were observed for *SPDYC*, *AP003774.1*, and *SNX15*, whereas controls showed distinct regulatory peaks for *ZNHIT2*, *BATF2*, and *EFEMP2*. **c** At an NK cell locus on chromosome 22, POAG-specific eQTLs were detected for *SSTR3* and *TEX33*, while control samples exhibited regulatory associations with *CDC42EP1*, *LGALS2*, and *KCNJ4*. These findings underscore the context-dependent nature of genetic regulation at POAG risk loci, with distinct sets of target genes and regulatory variants in disease versus control states.

**
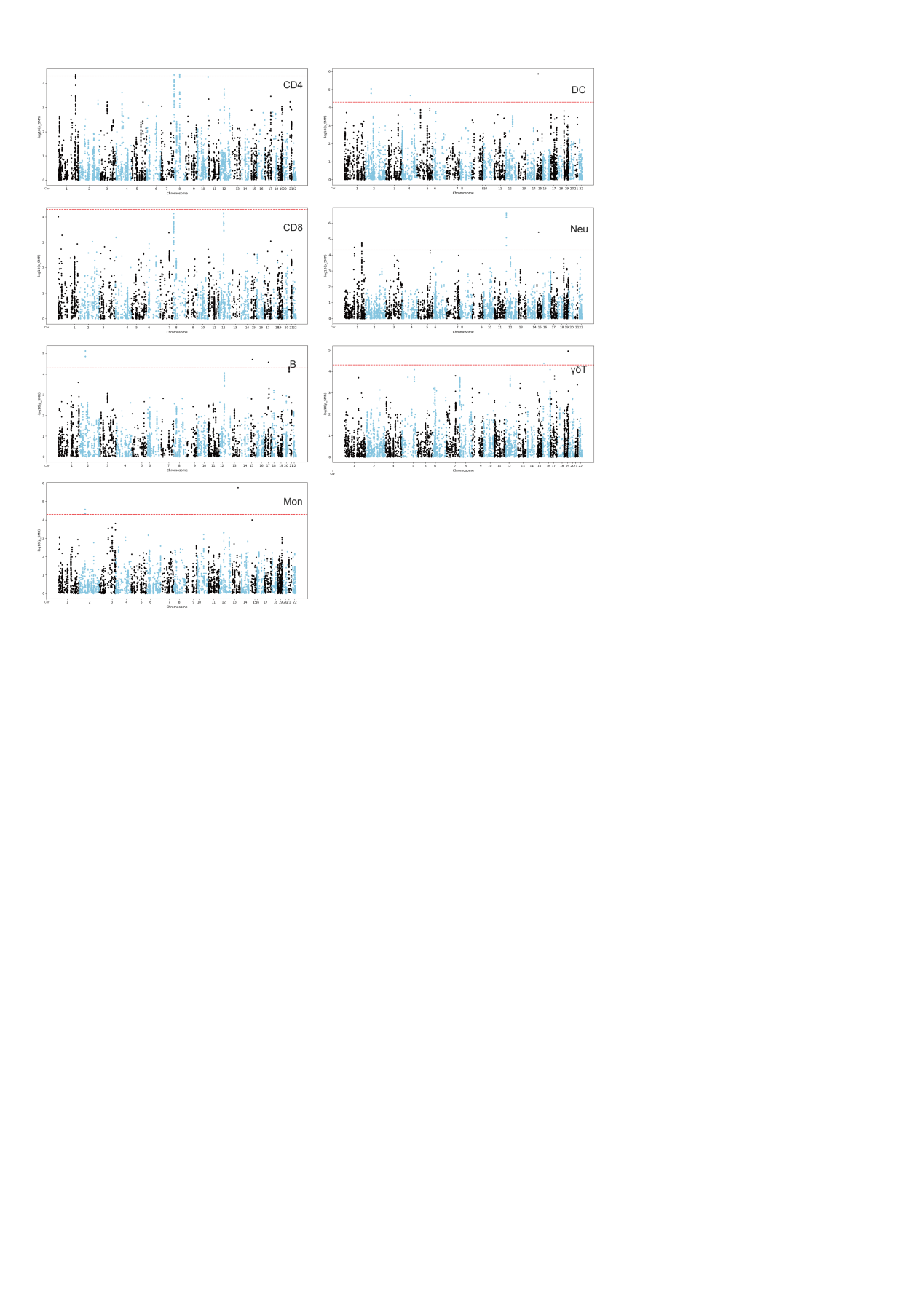
**

**Supplementary Fig. 9. SMR results at single-cell resolution for POAG**

The Manhattan plot of the cis-eQTLs in each of the celltypes in all samples, POAG patients, and healthy controls, respectively. Associations are reported as –log_10_(*P* value) (y-axis) ordered by chromosomes (x-axis).


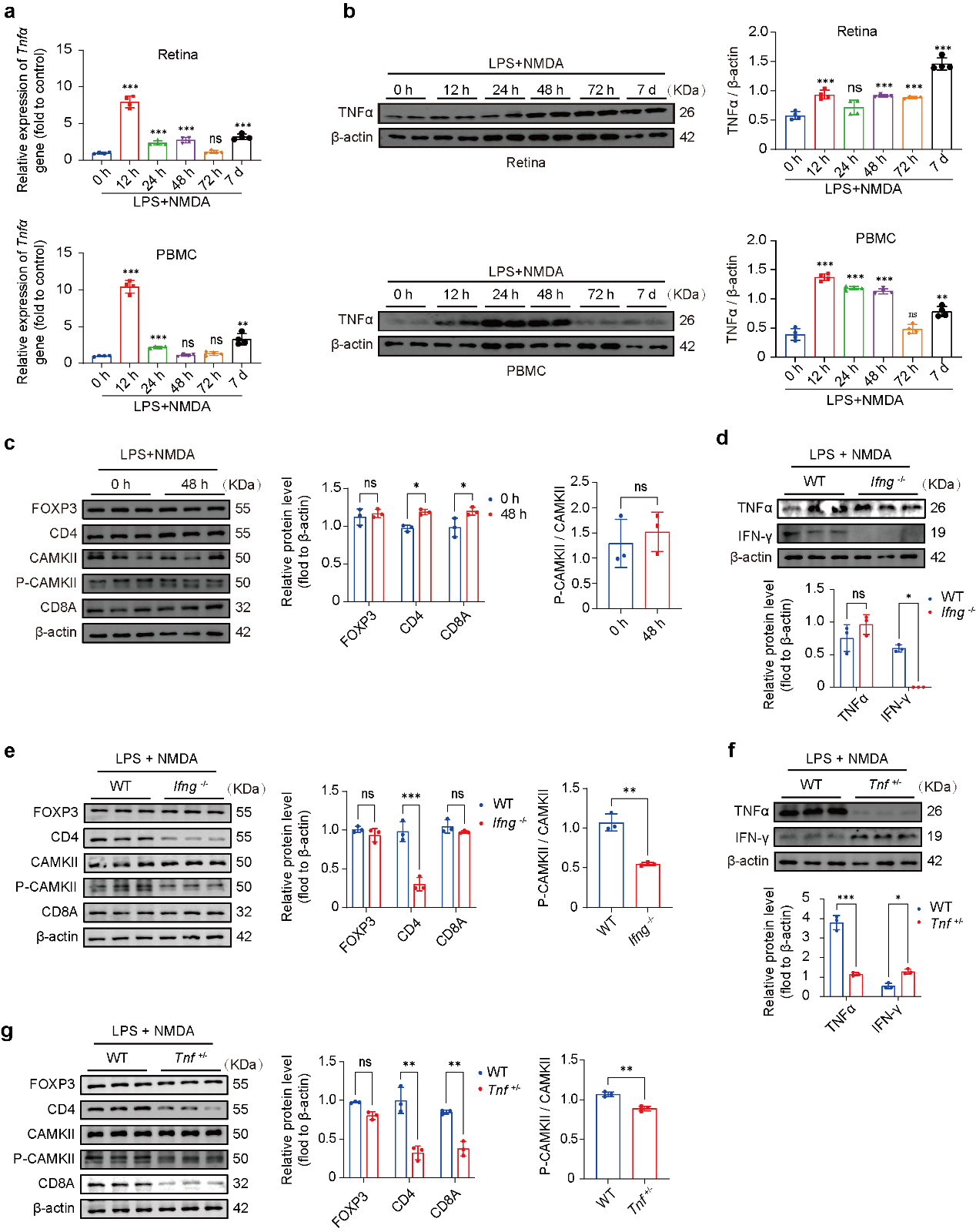


**Supplementary Fig. 10. *Ifng* ^-/-^ and *Tnf* ^+/-^ mice cause down-regulation of CD4+T and CD8+T cells. a** The expression of *Tnf* gene in the retina and PBMC of wild-type mice treated with LPS/NMDA was detected at each stage. **b** The expression of TNF in the retina and PBMC of wild-type mice treated with lps and NMDA was detected at each stage. **c** Wild-type mice were stimulated with Lps and NMDA for 0 and 48 hours respectively, and the expressions of CaMKII, P-CaMKII and various immune cells (FOXP3, CD4, CD8A) were detected by Western blotting in the retina. **d** *Ifng* ^-/-^ mice were stimulated by LPS/NMDA for 48 hours, and IFN-gamma and TNF were expressed in the retina. **e** The expression of CaMKII, P-CaMKII and immune cells (FOXP3, CD4, CD8A) in the retina of *Ifng* ^-/-^ mice 48 hours after stimulation with Lps and NMDA. **f** *Tnf* ^+/-^ mice were stimulated by LPS/NMDA for 48 hours, and IFN-gamma and TNF were expressed in the retina. **g** *Tnf* ^+/-^ mice were stimulated by LPS and NMDA for 48 hours, and IFN-gamma and TNF were expressed in the retina. Data presented as mean ± SEM. Statistical significance determined by unpaired Student’s t-test (n=3, per group) (*: *P < 0.05*, **: *P < 0.01*, ***: *P < 0.001*).


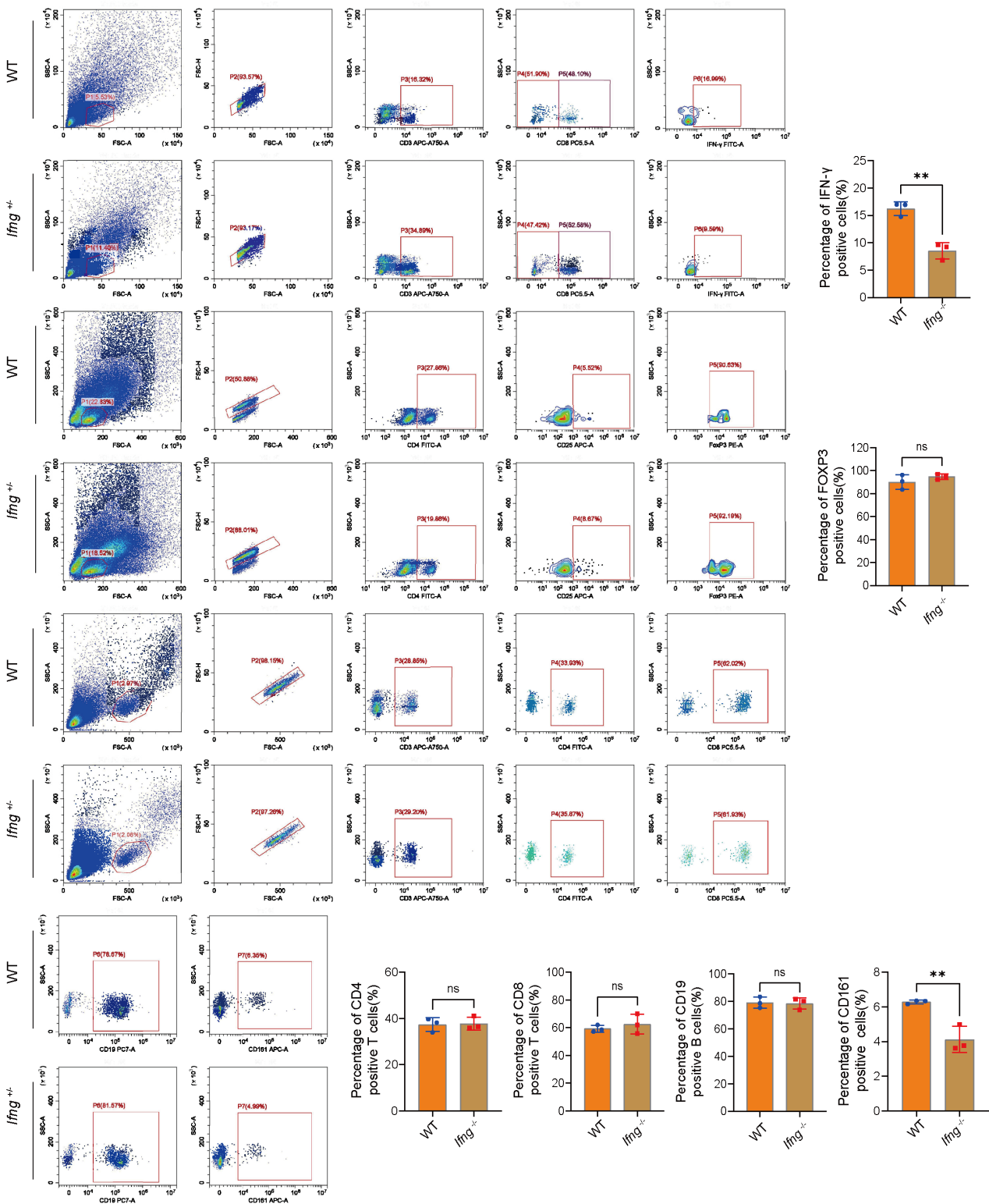


**Supplementary Figure 11: Peripheral immune cell populations in *Ifng* ^-/-^ mice following retinal injury.** Flow cytometry analysis of major immune cell populations in peripheral blood from WT and *Ifng* ^-/-^ mice 48 hours after LPS/NMDA treatment. Quantification of the percentage of CD4⁺ T cells, CD8⁺ T cells, B cells (CD19⁺), regulatory T cells (Tregs; Foxp3⁺), and NK cells (CD3⁻NK1.1⁺/CD161⁺) within the lymphocyte gate. *Ifng* ^-/-^ mice showed no significant changes in CD4⁺ T cells, CD8⁺ T cells, B cells, or Tregs, but exhibited a significant decrease in the percentage of NK cells compared to WT controls. Data are presented as mean ± SEM (n = 3). **：*P < 0.01* (unpaired Student's t-test).


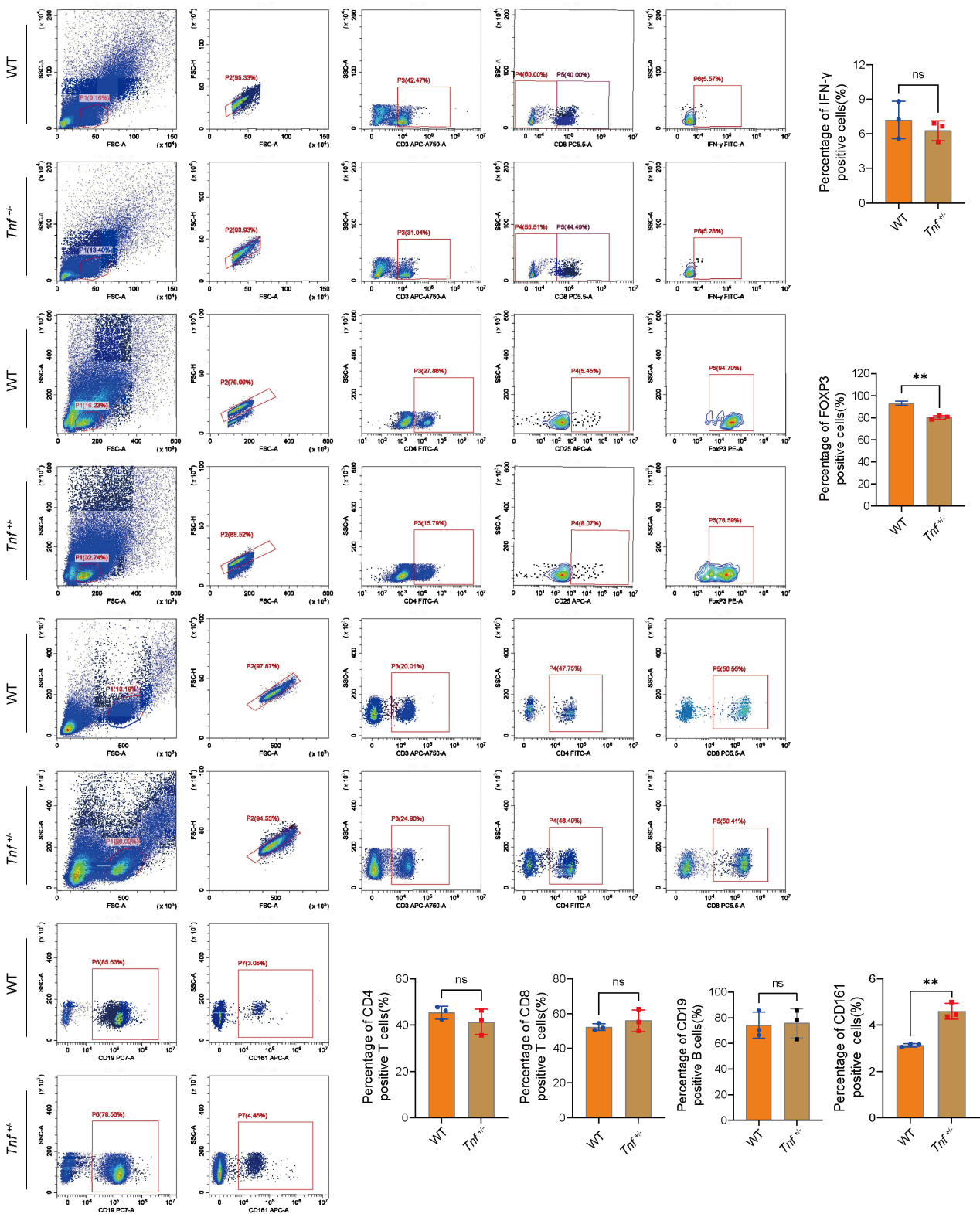


**Supplementary Figure 12: Peripheral immune cell populations in *Tnf* ^+/-^ mice following retinal injury.** Flow cytometry analysis of major immune cell populations in peripheral blood from WT and *Tnf* ^+/-^ mice 48 hours after LPS/NMDA treatment. Quantification of the percentage of CD4⁺ T cells, CD8⁺ T cells, B cells (CD19⁺), regulatory T cells (Tregs; Foxp3⁺), and NK cells (CD3⁻NK1.1⁺/CD161⁺) within the lymphocyte gate. *Tnf* ^+/-^ mice showed no significant changes in CD4⁺ T cells, CD8⁺ T cells, or B cells, but exhibited a significant decrease in the percentage of Tregs and a significant increase in the percentage of NK cells compared to WT controls. Data are presented as mean ± SEM (n = 3). **：*P < 0.01* (unpaired Student's t-test).

**
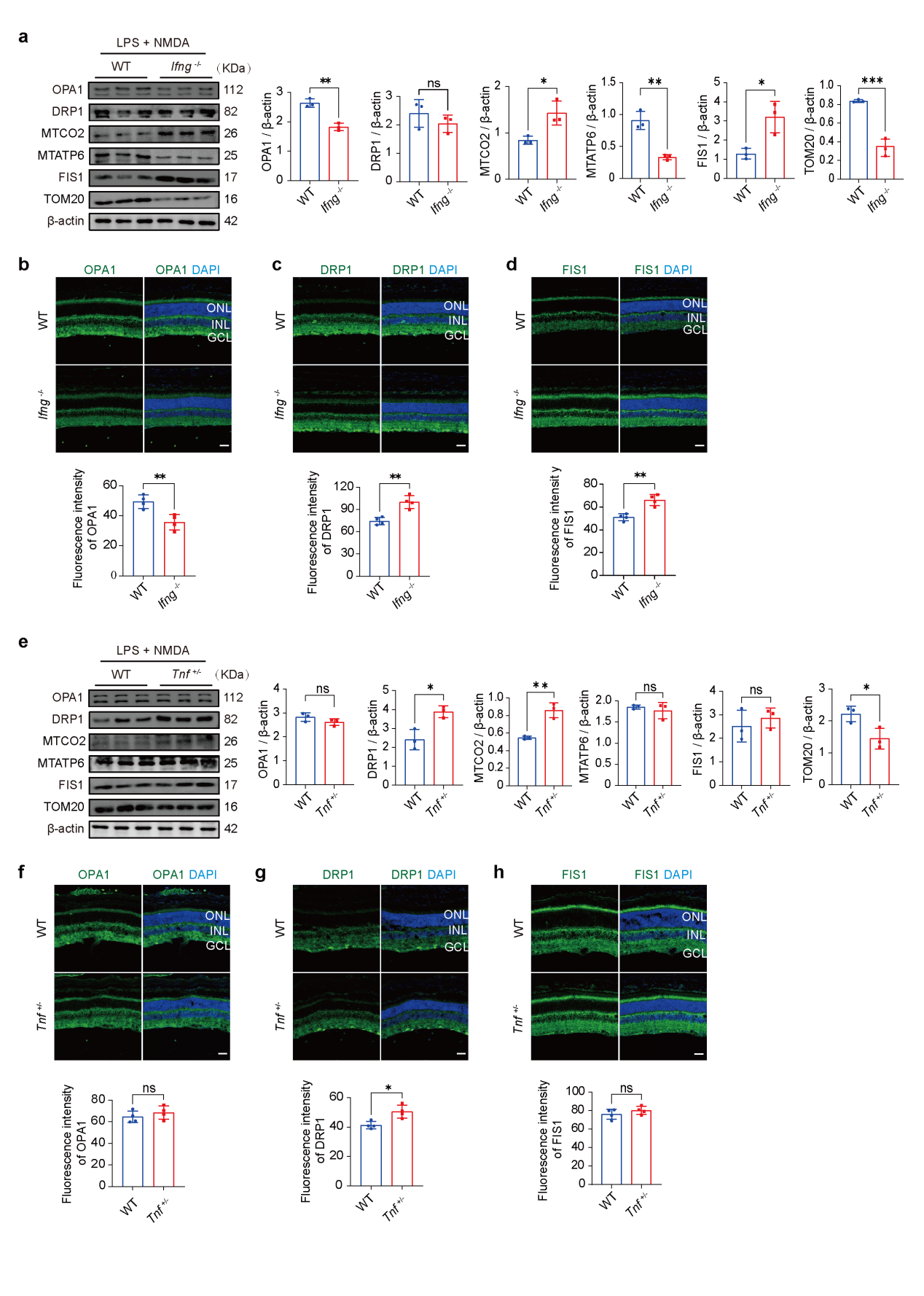
**

**Supplementary Fig. 13. *Ifng* ^-/-^ and *Tnf* ^+/-^ mice aggravated mitochondrial damage in the LPS/ NMDA-induced injury model. a** After 48 hours of LPS/NMDA induction in *Ifng* ^-/-^ mice, the expression of mitochondrial damage markers (OPA1, DRP1, MTCO2, MTATP6, TOM20, FIS1) in the retina was detected by Western blotting (n=3 WT, *Ifng* ^-/-^ per group). **b**-**d** Representative retinal cryosections and quantification of fluorescence intensity for: **b** OPA1(Mitochondrial fusion). **c** DRP1(Mitochondrial fission). **d** FIS1(Mitochondrial fission). (n=4 WT, *Ifng* ^-/-^ per group). Scale bar: 20 µm. **e** After 48 hours of LPS/NMDA induction in *Tnf* ^+/-^ mice, the expression of mitochondrial damage markers (OPA1, DRP1, MTCO2, MTATP6, TOM20, FIS1) in the retina was detected by Western blotting (n=3 WT, *Tnf* ^+/-^ per group). **f**-**h** Representative retinal cryosections and quantification of fluorescence intensity for: (f)OPA1(Mitochondrial fusion). **g** DRP1(Mitochondrial fission). **h** FIS1(Mitochondrial fission). (n=4 WT, *Tnf* ^+/-^ per group). Scale bar: 20 µm. Data presented as mean ± SEM. Statistical significance determined by unpaired Student’s t-test (*: *P < 0.05*, **: *P < 0.01*, ***: *P < 0.001*).

**References**

1. Wolf, F.A. *et al.* PAGA: graph abstraction reconciles clustering with trajectory inference through a topology preserving map of single cells. *Genome Biol* **20**, 59 (2019).

2. Wang, H. *et al.* A correlation-based feature analysis of physical examination indicators can help predict the overall underlying health status using machine learning. *Sci Rep* **12**, 19626 (2022).

3. Jiang, T. *et al.* A comprehensive genetic variant reference for the Chinese population. *Sci Bull (Beijing)* (2024).

4. Taylor-Weiner, A. *et al.* Scaling computational genomics to millions of individuals with GPUs. *Genome Biol* **20**, 228 (2019).

5. Consortium, G.T. The GTEx Consortium atlas of genetic regulatory effects across human tissues. *Science* **369**, 1318-1330 (2020).

6. Zhu, Z. *et al.* Integration of summary data from GWAS and eQTL studies predicts complex trait gene targets. *Nat Genet* **48**, 481-7 (2016).

7. Chen, Y. *et al.* Common variants near ABCA1 and in PMM2 are associated with primary open-angle glaucoma. *Nat Genet* **46**, 1115-9 (2014).
